# Diffusion Tensor Imaging Detects Acute Pathology-Specific Changes in the P301L Tauopathy Mouse Model Following Traumatic Brain Injury

**DOI:** 10.3389/fnins.2021.611451

**Published:** 2021-02-24

**Authors:** Neha Soni, Rodrigo Medeiros, Khawlah Alateeq, Xuan Vinh To, Fatima A. Nasrallah

**Affiliations:** Queensland Brain Institute, The University of Queensland, St. Lucia, QLD, Australia

**Keywords:** traumatic brain injury, diffusion tensor imaging, transgenic, tau-hyperphosphorylation, astrocytes, microglia, fractional anisotropy, P301L-mutation

## Abstract

Traumatic brain injury (TBI) has been linked with tauopathy. However, imaging methods that can non-invasively detect tau-protein abnormalities following TBI need further investigation. This study aimed to investigate the potential of diffusion tensor imaging (DTI) to detect tauopathy following TBI in P301L mutant-tau-transgenic-pR5-mice. A total of 24 9-month-old pR5 mice were randomly assigned to sham and TBI groups. Controlled cortical injuries/craniotomies were performed for TBI/sham groups followed by DTI data acquisition on days 1 and 7 post-injury. DTI data were analyzed by using voxelwise analysis and track-based spatial statistics for gray matter and white matter. Further, immunohistochemistry was performed for total-tau and phosphorylated-tau, astrocytes, and microglia. To detect the association of DTI with these pathological markers, a correlation analysis was performed between DTI and histology findings. At day 1 post-TBI, DTI revealed a widespread reduction in fractional anisotropy (FA) and axial diffusivity (AxD) in the TBI group compared to shams. On day 7, further reduction in FA, AxD, and mean diffusivity and increased radial diffusivity were observed. FA was significantly increased in the amygdala and cortex. Correlation results showed that in the ipsilateral hemisphere FA reduction was associated with increased phosphorylated-tau and glial-immunoreactivity, whereas in the contralateral regions, the FA increase was associated with increased immunostaining for astrocytes. This study is the first to exploit DTI to investigate the effect of TBI in tau-transgenic mice. We show that alterations in the DTI signal were associated with glial activity following TBI and would most likely reflect changes that co-occur with/without phosphorylated-tau. In addition, FA may be a promising measure to identify discrete pathological processes such as increased astroglia activation, tau-hyperphosphorylation or both in the brain following TBI.

## Introduction

Traumatic brain injury (TBI) has recently been deemed a leading risk factor for dementias such as Alzheimer’s disease (AD) or chronic traumatic encephalopathy (Fleminger et al., 2003; Hay et al., 2016; Li et al., 2017). People sustaining a TBI are 24% more likely to develop dementia, which increases with the severity and number of injuries (Fann et al., 2018). Distinctive but overlapping features of TBI and AD, apart from the associated cognitive deficits, are the deposition of fragments of the β-amyloid peptide to form plaques, and aggregation of hyperphosphorylated forms of the microtubule-associated protein tau to form intracellular neurofibrillary tangles (NFTs) (Tran et al., 2011a, b; Johnson et al., 2012; Magnoni et al., 2012). Clinicopathological studies show that NFTs are associated with brain function loss and the cognitive deficits reported in TBI and AD with the density of NFTs reflecting the degree of dementia (Yoshiyama et al., 2005; Johnson et al., 2016; Buckley et al., 2017). These findings indicate that tau is a valuable marker for the diagnosis of the long-term sequelae of TBI (Johnson et al., 2012). Immunohistochemical and biochemical methods have remained the gold standards for the detection of tau pathology in clinical, as well as experimental studies. In 5 to 7-month-old mice expressing wild-type human tau, accelerated tau pathology in the hippocampus, progressive astrogliosis, cognitive, and locomotor impairments were identified 6 weeks post-injury (Zhang et al., 2019). In a 3×Tg-AD mouse model overexpressing human tau and β-amyloid, accelerated hyperphosphorylation of tau and tau aggregates were observed 1 week after a single TBI (Tran et al., 2011a). Although both clinical and pre-clinical studies have proposed tau as a potential marker linking TBI to AD, the evidence has been largely based on **post mortem** evaluations and cannot be implemented in clinical settings. Moreover, the structural and pathological features of tauopathy have made its detection challenging even with **ex vivo** tools, hindering a concrete conclusion regarding its role in the link between TBI and AD (Hanger et al., 2009; Castellani and Perry, 2019). Therefore, there is a need to develop some **in vivo** tools to detect tau pathology at the early stages of disease in order to determine its effects during an individual’s lifespan.

Diffusion tensor imaging (DTI) is a magnetic resonance imaging (MRI) modality that uses magnetic field gradients to detect diffusivity parameters of water molecules as they undergo diffusion in biological tissues; and these diffusivities may be affected by pathological processes (Beaulieu, 2002; Alexander et al., 2007). Tau protein is soluble and responsible for microtubular stability. In the hyperphosphorylated state, it begins to aggregate in the form of insoluble paired helical filaments, and water mobility on the surface of the tau protein is highly affected in its transition from monomeric to aggregated forms (Fichou et al., 2015). Reduced fractional anisotropy (FA) and increased MD have also been reported in hippocampal regions of early AD patients; however, no studies were performed to understand the pathological underpinnings of these changes (Rose et al., 2008). In the rTg4510 tau transgenic mouse model, a strain with massive tau expression and neurodegeneration, reduced FA and increased radial diffusivity (RD) were noticed in white matter regions with tau aggregates (Sahara et al., 2014; Wells et al., 2015). In the triple transgenic 3×Tg mouse model of AD, decreased FA and axial diffusivity (AxD) were associated with the depositions of both amyloid-β and hyperphosphorylated tau in the hippocampal region (Snow et al., 2017). Although, DTI has been shown to detect hyperphosphorylated tau in AD patients and experimental models, no study is available that has investigated the potential of this method to detect tau abnormalities following severe TBI. Given that TBI involves a complex pathology and survivors can develop tauopathy within a week (Tran et al., 2011a; Rubenstein et al., 2017), it is crucial to understand if and how DTI reflects tau hyperphosphorylation in other confounding pathologies associated with brain injuries at its early stages.

Neuroinflammation is a crucial intrinsic process that has been implicated in the pathological events of tau aggregation and can be robustly induced by TBI (Johnson et al., 2013; Bemiller et al., 2017; Laurent et al., 2018; Pischiutta et al., 2018). Active glial cells (astrocytes and microglia) can increase tau hyperphosphosphorylation, followed by its aggregation (Leyns and Holtzman, 2017; Laurent et al., 2018). DTI has been shown to detect post-TBI neuroinflammation (Budde et al., 2011; Soni et al., 2018), but in AD, neuroinflammation has mostly been investigated using positron emission tomography imaging (Lagarde et al., 2018). Recently, Wang et al. (2015) proposed that DTI can detect inflammation in pre-clinical stages of AD by identifying reduced diffusivity measures (AD, MD, and RD). Thus, the evaluation of DTI to detect tau-induced structural brain abnormality at the early stages of TBI and to differentiate them from neuroinflammation-like pathologies can put more insight into DTI’s detection potential in TBI.

This work applies a DTI-histopathological approach to examine the effect of TBI on tau pathology progression in both white and gray matter structures in pR5 mice. pR5 mice overexpress the human tau isoform with a P301L mutation and a well-developed model to study tauopathy (Gotz et al., 2001). Since tauopathy is a key process that is triggered following TBI in the AD brain, confirmed both biochemically and histopathologically, we demonstrate, for the first time, the potential of DTI to map the spatial and temporal profile of the pathological processes following TBI in a tau transgenic mouse model.

## Materials and Methods

### Animals and Study Design

Twenty-four 9-month-old male and female P301L tau transgenic mouse (line pR5) (21–35 g) generated on a C57B1/6 × DBA2F1 background, and backcrossed onto C57B1/6 were used (Gotz et al., 2001). Transgenic mice were randomly allocated into two TBI and two sham groups at two respective time points, i.e., days 1 and 7 post-injury. To ensure the validity and precision of the statistical analysis, we used six mice per group. This study was approved by the Animal Research Ethics Committee of the University of Queensland (animal ethics committee number: QBI/SCMB/036/16/MAIC). All the experiments were performed in accordance with the Australian code of practice for the care and use of animals for scientific purposes.

**FIGURE 1:**
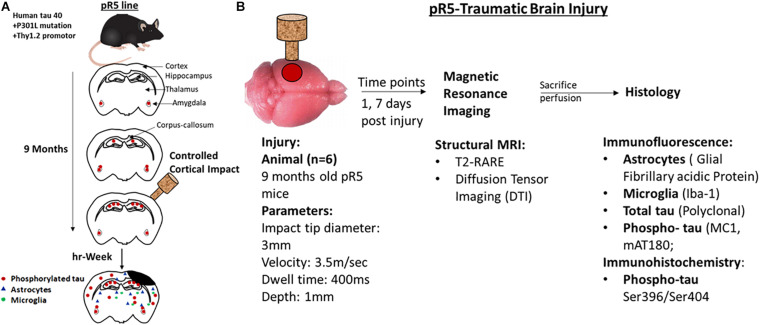
Representation of experimental design and experiments performed at different time points. **(A)** A schematic representation of the pathological progression in pR5 mice before and after injury in 9-month-old mice. **(B)** Experimental design, 9-month-old pr5 mice (*n* = 6/group) were exposed to controlled cortical impact followed by structural MRI scans at days 1 and 7, followed by histological analysis for astrocytes, microglia, total tau, and phospho-tau.

All animals were housed in the animal facility of the Center for Advanced Imaging, UQ, and acclimatized for a week before commencing surgeries. The TBI groups received a controlled cortical impact (CCI), whereas the sham groups underwent only craniotomy without an impact followed by MRI scan at the respective time points. All animals were immediately perfused after the MRI scans for histological studies. The study design is illustrated in Figure 1.

### Controlled Cortical Impact Model of Traumatic Brain Injury

Focal open skull injuries were performed using a CCI injury model following the same protocol described in our previous paper (Soni et al., 2018). Briefly, animals were anesthetized with 1.5–1.8% of isoflurane in a mixture of compressed air and oxygen (1:0.8) and fixed in a stereotactic frame with ear bars and a bite bar. Eye gel was applied to the eyes to avoid dryness. Craniotomy was performed on the left side of the brain, and the bone flap was removed from the craniotomy region with the dura intact. All mice in the TBI groups were exposed to a cortical impact using the CCI device (model-TBI 0310; Precision System and Instrumentation LLC.) with a 3 mm diameter piston tip. The brain was hit with an impact velocity = 3.5 m/s, depth = 1 mm, and dwell time = 400 ms, whereas, in the sham groups, only craniotomy was performed. In all cases, the bone flap was replaced, and the skin was sutured after the procedure in all mice. Animals were removed from the stereotaxic frame and placed on a heating pad for recovery. 0.5 ml of saline was injected subcutaneously in three different regions in all the animals for rehydration. All the mice were active within 5–10 min following injury and were kept in cages with *ad libitum* access to water gel and wet food.

### MRI Data Acquisition

All animals were handled for a minimum of 2 weeks prior to the scan. Animals were conditioned to handling for 5–10 min each day for a week before the surgery. On the day of surgery, each mouse was handled for 3–5 min before being placed in the anesthetic chamber, and then they were handled each day until the day of scanning. On the day of the scan, animals were also handled for 3–5 min before the induction of anesthesia to minimize stress. All TBI and sham animals underwent MRI scanning on days 1 and 7. MRI scans were performed on a 9.4T MRI scanner (Bruker BioSpin, Germany) equipped with a cryogenically cooled transmit and receive coil, controlled by a console running Paravision 6.0.1 (Bruker BioSpin, Germany) Deep anesthesia was induced by 3% of isoflurane in a mixture of compressed air/O_2_ (0.6/0.4) at 1 l/min, followed by 1.5–2% of isoflurane for maintenance. Deeply anesthetized rodents were then placed on an MRI-compatible cradle (Bruker Biospin) in the head first, supine position, and the head was fixed using ear bars and a bite bar to avoid movement. The respiration rate and rectal temperature were monitored by an MRI-compatible rodent physiological monitoring system (Model 1030, SA Instruments Inc.). Three−dimensional (3D) T2-weighted (T2w) imaging data sets were acquired using a rapid acquisition with relaxation enhancement (RARE) sequence with the following parameters: repetition time (TR) = 7200 ms, echo time (TE) = 39 ms, averages = 4, slice thickness = 0.3 mm, field of view (FoV) = 19.2 × 19.2 × 15.6 mm, and matrix = 192 × 192 × 52 totaling to 11 min 31 s of acquisition time/animal. DTI data were acquired using an axial gradient echo-planar imaging sequence using TR = 10,000 ms, TE = 25 ms, averages = 2, number of slices = 48, slice thickness = 0.3 mm, FoV = 18 × 18 × 15.6 mm, and matrix = 100 × 100 × 48, 2 b0 volumes, 33 non−collinear directions with *b*−value 750 s/mm^2^ with acquisition time of 11 min 40 s of acquisition time/animal. A reverse-phase image was acquired for DTI, which was used in data pre-processing.

### MRI Data Analysis

T2-weighted and DTI data sets were analyzed using the FMRIB Software Library version 5.0.9 (FSL)^1^. First, all T2w images were corrected for motion using the MCFLIRT tool (FMRIB’s Software Library) (Smith et al., 2004), followed by inhomogeneity correction using the N4BiasFieldCorrection function of the advanced normalization tools (ANTs Version: 2.1.0-gGIT-N). Corrected T2w datasets were then skull-stripped using brain masks created with the 3D Pulsed-Couple Neural Networks tool (Chou et al., 2011) and brain images were extracted. Skull-stripped individual T2w images were affinely registered to the Australian Mouse Brain Mapping Consortium template^2^ using FMRIB’s FLIRT. The registered images were averaged to create the first iteration of a study-specific template. This first iteration study-specific template was used as reference for non-linear registration of individual images using FMRIB’s FNIRT. The non-linearly registered individual images were averaged to generate the final study specific template to which a final round of non-linear registration of individual T2w images was performed.

Firstly, we used the reverse phase encoding volume pair as input for TOPUP (Andersson et al., 2003) to estimate distortion then correct all DWIs, then the MCFLIRT tool (FMRIB’s Software Library) (Smith et al., 2004) was used to adjust for eddy current distortion. The DTI data were then corrected for signal inhomogeneity using N4 Field bias correction from ANTS (ANTs Version: 2.1.0-gGIT-N). The FSL-diffusion Toolkit (DTI-FIT) (Behrens et al., 2003) was used for local fitting of the diffusion tensors to generate the maps for the DTI parameters FA, MD, AxD, and RD using *b*-values 0 and 750 s/mm. To register the data to the study specific template, we firstly co-registered the individual’s B0 maps to the corresponding T2 images and then apply the warp files from T2-to-study template to register the DTI measures to the same common space of study specific template. All estimated DTI maps were registered to the study-specific T2w template for inter-group voxelwise permutation testing.

To improve the sensitivity, objectivity, and interpretability of the white matter changes, voxel-based analysis style-TBSS (track-based spatial statistics) was performed by using the standard pipeline in FSL, FMRIB (Smith et al., 2006, 2007). In this analysis method, all FA maps in a common study−specific T2w template space were first averaged to create the mean FA template, followed by generation of the mean skeletonized FA maps (representation of fiber tracts). The individual skeletonized mean FA map was further used to generate a distance map. The distance map, together with the FA map thresholded at 0.2 and the anterior commissure as the reference, was used to get a TBSS skeleton for the individual FA maps and subsequently for all other individual metrics (AxD, RD, and MD skeletonized) to show changes in the white matter tracks. The mean FA template was used as an underlay to represent the diffusion changes in the white matter. A demonstration of DTI image registration efficiency and TBSS skeletonization procedure can be found in Supplementary Figures 1, 2. Which demonstrated the efficiency of image registration, its short comings with regards to larger white matter tracts while acceptable for large homogenous gray matter structures, and TBSS efficiency and accuracy in improve the matching across subjects and reflect, for example, missing white matter tracts in the lesion area.

For region-of-interest based analyses, we mainly focused on the regions with maximal changes seen on statistical difference maps obtained from the voxel-based analysis. These regions involved the corpus callosum (middle region), ipsilateral (ip) and contralateral (cn) external capsule, and the internal capsule. In the gray matter, the cn-dentate gyrus, CA1 region (the ipsilateral hippocampus was washed away during brain processing for histology), ipsilateral and contralateral amygdala, cortex, and thalamus were explored. Regions-of-interest were drawn manually on the study-specific template using the Australian mouse brain mapping consortium template (Watson et al., 2017) and the Allen adult mouse brain atlas^3^ as a reference (Soni et al., 2020). Diffusion and anisotropy values were extracted from the regions-of-interest and correlated with the measures from immunohistochemistry, namely, phosphorylated tau, microglia, and astrocytes.

### Histology Preparation and Analysis

Immediately after MRI scans, the mice were deeply anesthetized with isoflurane and perfused transcardially with 11 ml 0.1 M phosphate-buffered saline containing 1% sodium nitrite (PBS, pH 7.4) followed by 11 ml 4% formaldehyde (prepared from paraformaldehyde) dissolved in 0.1 M PBS. Perfusates were delivered using an automated syringe pump [10 ml–14.48 mm diameter BD (New Jersey, United States) syringes were used] programed to deliver perfusates at 90 ml/h. Brains were excised and immersion-fixed in 4% PFA overnight at 4°C and then stored in PBS supplemented with 0.05% sodium azide. Prior to slicing, the brains were cryoprotected with 30% sucrose for 48 h. The frozen brains were sectioned coronally (40 μm thick) using a Leica SM2010R freezing microtome and collected serially in PBS with 0.02% sodium azide and kept at 4°C until used for immunohistology. In accordance with the 3rd edition of the Franklin and Paxinos, 1997 mouse brain atlas, five coronal mouse brain sections were selected (between Bregma −0.5 and −4.5 mm) for each immunohistology staining (Every 25th coronal section of 40 μm thickness). Allen mouse brain atlas (see text footnote 3) was also referred to cross verify regions of interest for clarity.

### Immunohistochemistry

For immunohistochemistry, free-floating brain sections were rinsed thrice with tris-buffer saline (TBS, pH 7.5) and then incubated with 30% H_2_O_2_ in methanol in TBS for 30 min to quench the endogenous peroxidase activity. This was followed by TBS washing and citrate buffer treatment at 95°C for 10 min for antigen retrieval. Sections were then treated in 0.1% Triton X-100 in TBS (TBS-A; pH = 7.5) for 15 min, followed by blocking in 2% bovine serum albumin in TBS and Triton X-100 (TBS-B; pH = 7.5) for 30 min. Brain sections were further incubated with the primary antibody for phosphorylated-tau [rabbit pSer (396 + 404) (Thermo Fisher; 1:1,000)] with 5% normal-horse-serum and kept on the shaker overnight at 4°C. The tissues were washed gently for 15 min the next morning. Prior to the secondary antibody treatment, sections were blocked with 5% normal-horse-serum diluted in TBS-B for 30 min and then incubated with secondary biotinylated horse anti-rabbit immunoglobulin-G (Vector Laboratories; 1:500) for an hour. After 20 min of blocking with TBS-B, the sections underwent Avidin-Biotin Complex (ABC) reagent treatment (Vector laboratories) for 30 min, followed by 3,3′-Diaminobenzidine (DAB) treatment with hydrogen peroxide at room temperature. The DAB treatment time was strictly controlled to obtain comparable results. The sections were then rinsed with TBS and mounted on superfrost-plus slides. After drying, the slides were cover-slipped with the mounting medium and allowed to dry overnight.

### Immunofluorescence

For total tau, immunofluorescence was performed. Except for H_2_O_2_ and citrate buffer treatment, the initial steps were similar to those used for immunohistochemistry. Brain sections were then incubated with primary antibody (polyclonal rabbit anti-human) (Dako; 1:1,000) for total tau. Goat anti-rabbit 488 (Life Technologies; 1:200) was used as the secondary antibody. The sections were rinsed, mounted, dried, and cover-slipped. Double immunofluorescence was performed for astrocytes and microglia using glial fibrillary acidic protein (GFAP) and ionized calcium-binding adaptor molecule 1 (Iba1) markers, anti-GFAP chicken polyclonal antibody (Abcam; 1:1,000) and anti-Iba1 rabbit monoclonal antibody (Wako; 1:1,000). Goat anti-rabbit 555 (Life Technologies; 1:200) and goat-anti chicken 488 (Life Technologies; 1:200) secondary antibodies were used.

### Quantification

Sections were examined for staining under an upright microscope (Axio Imager Green). For the quantification, slides were scanned using a Metafer VSlide scanner (MetaSystems) using a Zeiss Axio Imager Z2. Each section of the slides was then cropped into individual images using the 3D crop tool of the interactive microscopy image analysis software (Imaris; Bitplane). For the histology figures, selected areas of interests were acquired using a spinning-disk confocal system (Marianas; 3i, Inc.) consisting of an Axio Observer Z1 (Carl Zeiss), CSU-W1 spinning-disk head (Yokogawa Corporation), ORCA-Flash4.0 v2 sCMOS camera (Hamamatsu Photonics), using 63× magnification oil immersion objective. Images were acquired as 20 μm Z-stacks with 0.5 μm intervals and the 3D stacks were maximum intensity Z-projected to create a single 2D image for figures.

The percentage area of phosphorylated-tau positive immunoreactivity was calculated as described previously (Bodea et al., 2017). Total five serial sections per animal were used for the histological quantification. Using ImageJ, two sample boxes, one large and one small were created. These two sample boxes were utilized to create multiple ROIs of the same dimensions that were then placed on the respective areas of interest on the slices (corpus callosum, internal capsule, external capsule, cortex, dentate gyrus, CA1, thalamus, and amygdala). Please refer to Supplementary Figure 3 for the ROI details. These regions were chosen on the basis of voxel-based analysis results of DTI maps. To be consistent, same ROIs were used for all the animals with slight adjustments for the regions if needed. For total-tau, astrocyte and microglia sections were corrected for the background using the rolling ball algorithm, then thresholded using the automated ImageJ threshold (moment), where staining was not visible in the negative control. Cell number and percent area covered by cells were then calculated using the particle analyzer tool. Any region that has multiple boxes, average values were calculated. The graphs are platted statistically while the images shown are the representative images from a random subject of each group. Slight differences in the contrast in the representative images could be during data acquisition from the automatic scanning, however the data acquisition parameters were well optimized and consistent for the data. Visual analysis was performed to check the major deformations, link to the data repository can be provided if needed.

### Statistical Analysis

For voxel-based analysis, an unpaired two-sample *t*-test was used to calculate the differences between the TBI and sham animals for the DTI parameters using the FSL−randomize tool with the number of permutations set to exhaustive. The FSL-threshold-free cluster enhancement corrected difference maps, thresholded at *p*-value ≤ 0.05 results, were reported.

For region-of-interest-based analysis, prior to fitting the statistical models, we examined the normality of the data using the Shapiro–Wilk test. All the data sets were normally distributed, so parametric tests were used for the analysis. One-way ANOVA with *post hoc* analysis using Tukey’s multiple comparisons test was used. Data analysis was completed and plotted using GraphPad Prism version 7.04 (GraphPad Software Inc.). The results are presented as mean ± standard error measurement. ^∗^*p*-value ≤ 0.05, ^∗∗^*p*-value ≤ 0.001, and ^∗∗∗^*p*-value ≤ 0.0001 were considered significant.

In order to investigate the possible association between DTI measures, tau pathology, and neuroinflammation, Pearson’s multiple correlations were performed. The calculated *R*^2^ or the coefficient of correlation values and corrected *p*-values are reported in the results. A *p*-value ≤ 0.05 was considered significant. As we have *n* = 6, it was not enough separate the data and perform correlation within separate groups. Thus, both sham and TBI groups were combined for correlation.

## Results

In this study, the effects of TBI on the microstructure of the white and gray matter were examined by determining the associated changes of water diffusion using DTI, with corresponding pathological analyses in the same animals from histological specimens. Here, we report the whole brain voxel-based analysis results. Further, we discuss our immunohistochemistry findings and their correlation with DTI measures (FA, MD, AxD, and RD) in several white matter and gray matter regions selected on the basis of the voxel-based analysis results.

### Diffusion Tensor Imaging Reveals Changes in the White Matter of pR5 Mice Following TBI

**FIGURE 2:**
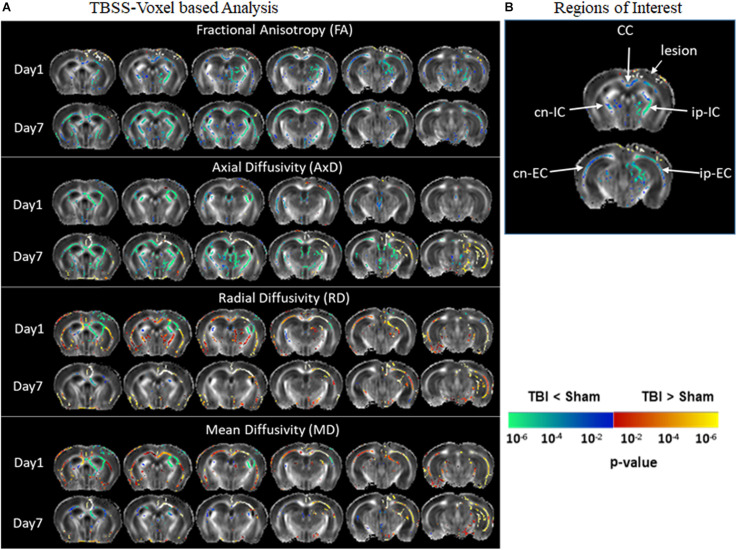
Alterations in DTI measures (FA, AxD, MD, and RD) in white matter representing statistical differences between pR5-sham and pR5-TBI groups over 1 week. **(A)** Tract-based spatial statistical Intergroup difference maps for DTI parameters fractional anisotropy (FA), axial diffusivity (AxD), mean diffusivity (MD), and radial diffusivity (RD) between sham (*n* = 6) and TBI groups (*n* = 6) at different time points overlaid on the FA-template (columns). Yellow–red indicates TBI > sham, and blue–green indicates TBI < sham. The statistical map was thresholded at *p*-value ≤ 0.05, unpaired two-sample *t*-test, implemented as permutation tested for the General Linear Model. **(B)** Showing the regions of interest chosen for the correlation analysis involving the corpus callosum, ipsilateral internal capsule, contralateral internal capsule, ipsilateral external capsule, and contralateral external capsule.

Figure 2 represents significantly affected white matter regions after TBI as compared sham controls. From the TBSS results (Figure 2A), FA was significantly low in pR5 mice following TBI compared to sham-treated pR5 mice in the white matter regions ipsilateral to the lesion side involving the corpus callosum, external capsule, fimbria, stria terminalis and internal capsule, optic tracks, and external medullary lamina of the thalamus on day 1 post-injury. FA reduction was also seen in the contralateral-external capsule of the pR5 TBI mice. On day 7, in addition to the above-mentioned regions, a significant reduction in FA was observed in the corpus callosum, fimbria, and internal capsule in the TBI mice compared to the sham mice. In the case of diffusivity changes, on day 1 post-injury, a significantly reduced AxD was noted in the ipsilateral and contralateral fimbria and external capsule in the TBI mice as compared to the sham mice, whereas RD and MD were significantly higher. A similar increase in RD and MD was observed in the corpus callosum, ipsilateral internal capsule, and contralateral internal capsule. On day 7 post-injury, a significant AxD reduction was evident in the corpus callosum and the contralateral fimbria, internal capsule, and external capsule regions in the TBI group versus the sham group. Unlike on day 1, RD and MD changes were negligible except in the ipsilateral external capsule, where increased RD and MD changes were noted in the TBI mice. These results indicate that DTI measures were potentially detecting the white matter abnormalities induced by TBI in tau transgenic mice.

### DTI Reveals Changes in the Gray Matter in pR5 Mice Following TBI

**FIGURE 3:**
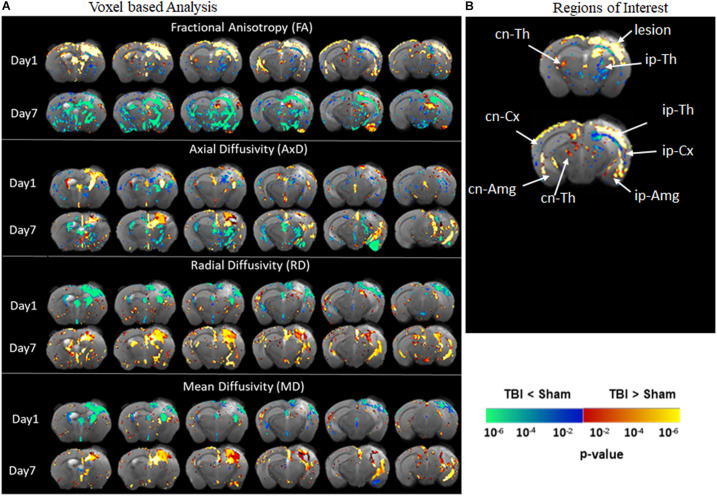
Alterations in DTI measures (FA, AxD, MD, and RD) in gray matter representing statistical differences between pR5-sham and pR5-TBI groups 1 week after TBI. **(A)** Voxel-by-voxel statistical analysis results of DTI parameters fractional anisotropy (FA), axial diffusivity (AxD), mean diffusivity (MD), and radial diffusivity (RD) between sham and TBI groups at different time points overlaid on the FA-template (columns). Yellow–red indicates TBI > sham, and blue–green indicates TBI < sham. The statistical map was thresholded at *p*-value ≤ 0.05, unpaired two-sample *t*-test, implemented as permutation tested for the General Linear Model. Fractional anisotropy changes were extremely high after 7 days post-injury in the gray matter. **(B)** Showing the regions of interest chosen in the correlation analysis, involving ipsilateral and contralateral thalamus, amygdala, cortex, and contralateral hippocampal regions. MRI regions of interest are also presented in Supplementary Figure 3B.

Diffusion tensor imaging results shown in Figure 3 reveal significant destruction in the gray matter microstructural regions. In voxel-based analysis findings (Figure 3A), on day 1 post-injury, there was a significant reduction in FA in the ipsilateral caudate-putamen and the thalamus in the TBI group as compared to the sham group. On day 7, together with the ipsilateral regions, a significantly reduced FA was apparent in the contralateral regions involving the caudate-putamen, cortex, and hippocampus (CA1 and dentate gyrus). The FA was significantly increased in the piriform amygdala, cortex, and in the deep gray matter in close proximity to the lesion. A significant reduction in AxD in the TBI group versus the sham group was mainly found in the ipsilateral and contralateral cortex, the ipsilateral thalamic area closer to the injury site, and in the caudal hippocampus on day 1 post-injury. On day 7, a widespread reduction in AxD in the TBI group as compared to the sham group was observed in the regions involving the ipsilateral caudate-putamen, thalamus, and amygdala. Unlike on day 1, a significant increase in the AxD was seen in the cortex and caudal hippocampus. The RD and MD were significantly increased in cortical and thalamic areas in the TBI group versus the sham group after day 1 post-injury. On day 7, significant increases in RD and MD were noted only in the cortex of TBI group as compare to sham group. Overall, these results indicate that TBI induced structural abnormalities in tau transgenic mice were significantly altering all DTI measures. Both increased and decreased FA changes were noted in different in gray matter regions.

### Increased Tau Phosphorylation Post-injury in pR5 Mice

**FIGURE 4:**
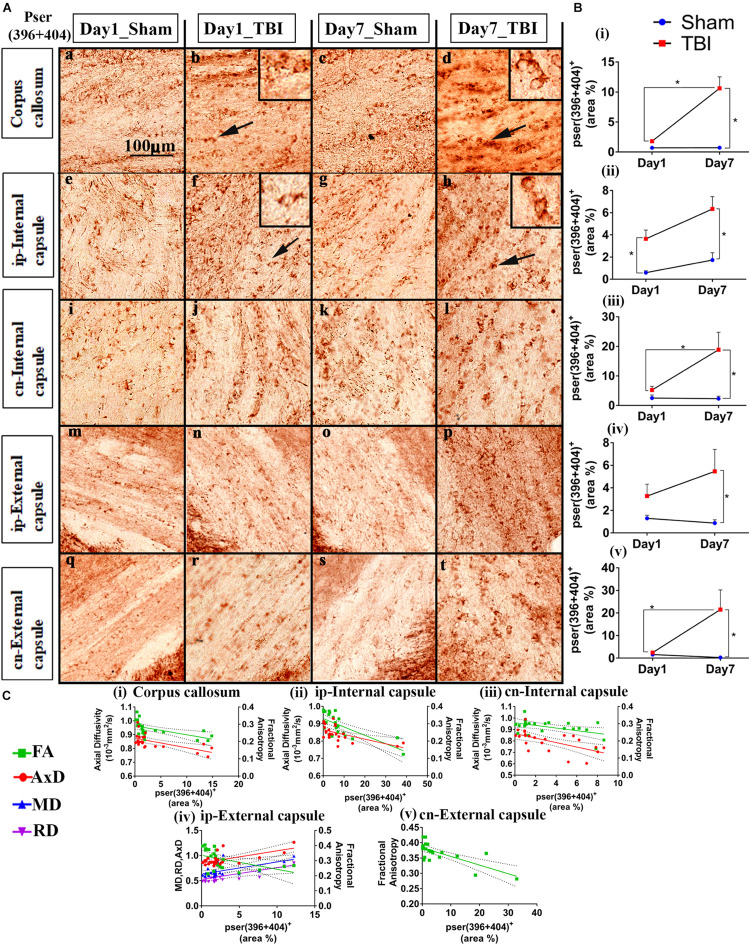
Controlled cortical impact increases phosphorylation in the white matter of injured pR5 mice: **(A)** Phosphorylation at the pSer396 + 404 epitopes of tau was detected in different white matter regions, including the corpus callosum (a–d), ipsilateral internal capsule (e–h), contralateral internal capsule (i–l), ipsilateral external capsule (m–p), and contralateral external capsule (q–t) of pR5 mice post-injury (*n* = 6 pR5 TBI mice and *n* = 6 pR5-sham mice at days 1 and 7). Scale bar: 100 μm. Zoom-in view of phospho-tau positive cells indicated with the black arrows are shown in the square boxes. **(B)** (i–v), Phosphorylation at the pSer396 + 404 epitopes was markedly increased in all the regions (corpus callosum, ipsilateral internal capsule, contralateral internal capsule, ipsilateral external capsule, and contralateral external capsule) on day 7 post-injury in the pR5-TBI group versus the pR5-sham group, whereas no significant differences were observed on day 1 pR5-TBI group versus the pR5-sham group except contralateral internal capsule (iii). One-way ANOVA with Tukey’s multiple comparison test, *p ≤ 0.05, ** *p* ≤ 0.001, ***p ≤ 0.0001). **(C)** (i–v), Anisotropy was significantly reduced with hyperphosphorylation in all regions (corpus callosum, ipsilateral internal capsule, contralateral internal capsule, ipsilateral external capsule, and contralateral external capsule). A negative correlation was also found with axial diffusion in corpus callosum, ipsilateral internal capsule, contralateral internal capsule, and contralateral external capsule whereas in ipsilateral external capsule axial diffusivity (AxD) was increased significantly with increased phosphorylation. Radial diffusivity (RD) and mean diffusivity (MD) were also increased markedly with phosphorylation in the ipsilateral external capsule (**C** (iv)) (Pearson multiple correlations *p*-value ≤ 0.05).

**FIGURE 5:**
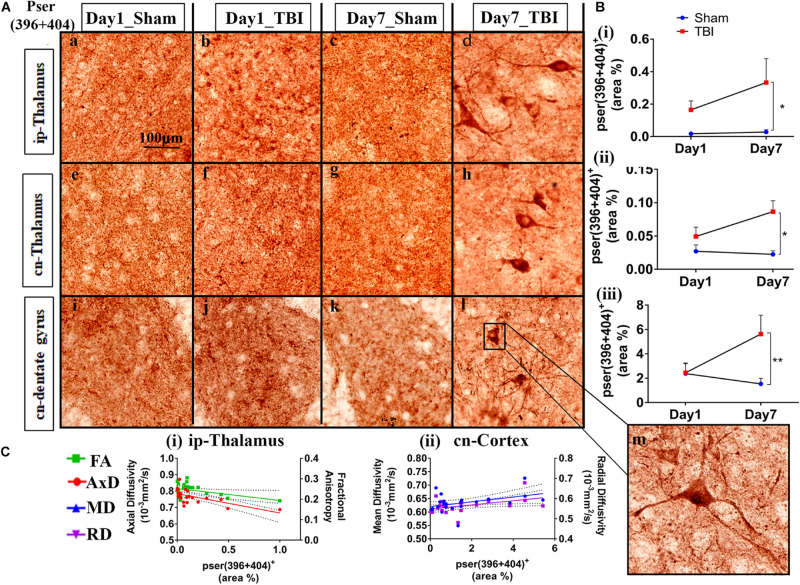
Phosphorylation occurred in additional gray matter regions 1-week post-TBI in injured pR5 mice: **(A)** Phosphorylation at the pSer396 + 404 epitopes of tau was detected in the ipsilateral thalamus (a–d), contralateral thalamus (e–h), and contralateral dentate gyrus (i–l) (*n* = 6 pR5 TBI mice and *n* = 6 pR5-sham mice at days 1 and 7). Scale bar: 100 μm. **(B)** (i–iii), Phosphorylation at the pSer396 + 404 epitopes was evidently increased in the regions on day 7 post-injury in the pR5-TBI group versus the pR5-sham group, whereas no significant differences were observed in the day 1 pR5-TBI group versus the pR5-sham group (one-way ANOVA with Tukey’s multiple comparisons test, *p ≤ 0.05, **p ≤ 0.001). **(C)** (i–ii), Fractional anisotropy (FA) and axial diffusivity (AxD) were significantly reduced with increased phosphorylation in the ipsilateral thalamus. Although hyperphosphorylation was not significant in the cortex, radial and mean diffusivity was significantly correlating with tau hyperphosphorylation in the cortex (Pearson multiple correlations *p*-value ≤ 0.05).

Immunohistochemistry revealed post-injury increase in tau hyperphosphorylation at the epitope site ser396 + 404 in several white and gray matter regions (Figures 4A, 5A). This increased pathology was potentially reflected by the various DTI measures as shown in our correlation analysis, represented in Figures 4C, 5C.

Statistically (Figure 4B), on day 1, a significant increase in the percentage of Ser396 + 404-positive area was observed in the cn-internal capsule in the TBI group (3.638 ± 0.7967; *p* = 0.0482) versus the sham group (0.5933 ± 0.1722). Immunoreactivity for phosphorylated tau was not altered in the ip-external capsule (3.271 ± 1.056; *p* = 0.6090), ip-internal capsule (5.254 ± 1.250; *p* = 0.9211), and corpus callosum (1.799 ± 0.0858; *p* = 0.8524) compared to the sham group; ip-external capsule (1.293 ± 0.2726), ip-internal capsule (2.514 ± 1.046), and corpus callosum (0.7057 ± 0.1883). On day 7, immunoreactivity for phosphorylated tau was significantly higher in all selected regions-of-interest in the TBI group [ip-external capsule (5.456 ± 1.951; *p* = 0.0426), ip-internal capsule (18.81 ± 5.868; *p* = 0.0057), cn-internal capsule (6.331 ± 1.119; *p* = 0.0020), and corpus callosum (10.61 ± 1.911; *p* = 0.0001)] as compared to the day 7 sham group [ip-external capsule (0.8712 ± 0.2962), ip-internal capsule (2.299 ± 0.8450), cn-internal capsule (1.733 ± 0.6496), and corpus callosum (0.7123 ± 0.1344)]. In the corpus callosum (10.61 ± 1.911; *p* = 0.0001), ip-internal capsule (18.81 ± 5.868; *p* = 0.0257), and cn-external capsule (21.51 ± 8.695; *p* = 0.0439), immunoreactivity for phosphorylated tau in the day 7 TBI group was even higher than in the day 1 TBI group [corpus callosum (1.799 ± 0.0858), ip-internal capsule (5.254 ± 1.250), and cn-external capsule (2.434 ± 0.4153)]. Notably, immunoreactivity for phosphorylated tau was more abundant in contralateral regions than ipsilateral regions. There was no significant increase in total tau levels (data not shown).

Upon correlation with the DTI measures (Figure 4C), a negative correlation was seen between the percentage of ser396 + 404 positive area and FA in the corpus callosum (*R*^2^ = 0.3330; *p* = 0.0039), ip-external capsule (*R*^2^ = 0.2269; *p* = 0.0216), ip-internal capsule (*R*^2^ = 0.5076; *p* = 0.0001), cn-external capsule (*R*^2^ = 0.5435; *p* = 0.0001), and cn-internal capsule (*R*^2^ = 0.2483; *p* = 0.0155). A negative correlation was also observed with AxD in the corpus callosum (*R*^2^ = 0.3654; *p* = 0.0022), ip-internal capsule (*R*^2^ = 0.3438; *p* = 0.0026), and cn-internal capsule (*R*^2^ = 0.3710; *p* = 0.0020). Interestingly, a positive correlation was noted in the ip-external capsule phosphorylated-tau and diffusivity measures of AxD (*R*^2^ = 0.3969; *p* = 0.0013), MD (*R*^2^ = 0.3509; *p* = 0.0029), and RD (*R*^2^ = 0.3004; *p* = 0.0068). Overall, these results indicate that decreased FA is associated with increased tau pathology.

In the gray matter (Figure 5B), no significant changes were observed on day 1 post-TBI. On day 7, a significant increase in the phosphorylated-tau positive area was seen only in the ip-thalamus (0.3324 ± 0.1478; *p* = 0.0488), cn-thalamus (0.08643 ± 0.01638; *p* = 0.0062), and cn-dentate gyrus (5.619 ± 1.554; *p* = 0.0318) in the TBI group versus the day 7 sham group [ip-thalamus (0.01743 ± 0.0055), cn-thalamus (0.02256 ± 0.0052), cn-dentate gyrus (1.533 ± 0.4619)]. In other regions involving the ip-cortex (3.099 ± 1.498; *p* = 0.4406), cn-cortex (2.353 ± 0.7649; *p* = 0.3659), cn-amygdala (4.896 ± 2.076; *p* = 0.5464), and cn-CA1 (18.13 ± 4,775; *p* = 2.447), a non-significant increase was noted as compared to the respective sham group [ip-cortex (0.6220 ± 0.3059), cn-cortex (0.9850 ± 0.3171), cn-amygdala (1.432 ± 0.6714), and cn-Ca1 (7.971 ± 3.573)]. These findings indicate that cn-dentate gyrus and ip- and cn-thalamus were the two main gray regions that were affected with tau pathology within a week in the pR5 mice after injury.

Similar to the white matter, a negative correlation of phosphorylated-tau area was observed with FA (*R*^2^ = 0.1865; *p* = 0.0396) and AxD (*R*^2^ = 0.3938; *p* = 0.0013) but only in the ip-thalamus. In the cn-cortex, a positive correlation was observed with MD (*R*^2^ = 0.1802; *p* = 0.0435) and RD (*R*^2^ = 0.2020; *p* = 0.0314) (Figure 5C). This suggests that a decrease in FA in the gray matter is associated with increased tau hyperphosphorylation.

### Increased GFAP Expression Post-injury in pR5 Mice

**FIGURE 6:**
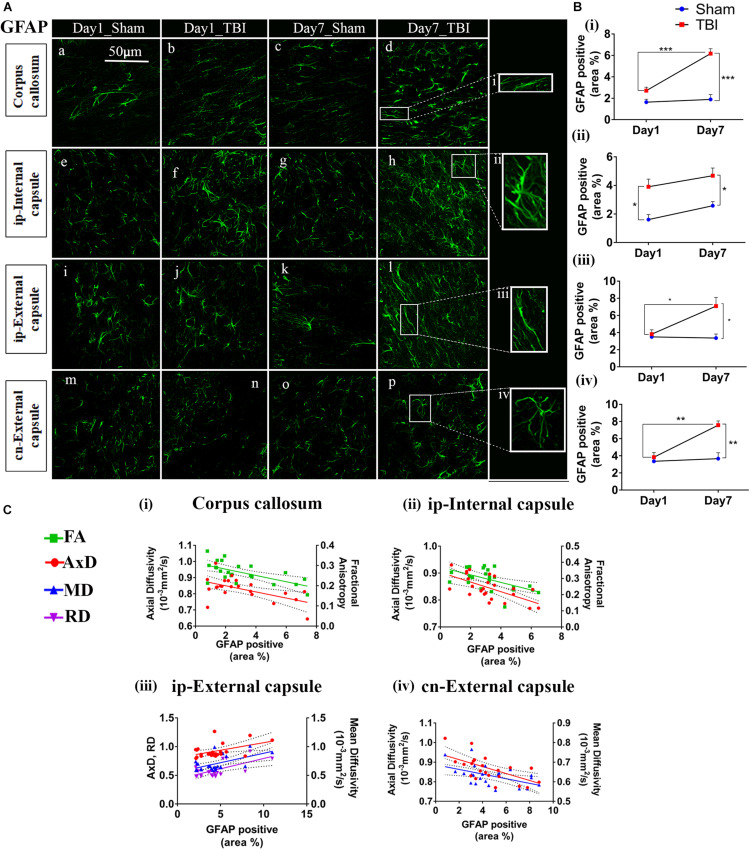
Increased astrogliosis in white matter early after injury in pR5 mice: **(A)** Increase in glial fibrillary acidic protein (GFAP) positive cell area percentage was noticed in white matter regions containing the corpus callosum (a–d), ipsilateral internal capsule (e–h), ipsilateral external capsule (i–l), and contralateral external capsule (m–p) of injured pR5 mice (*n* = 6 pR5 TBI mice and *n* = 6 pR5-sham mice at days 1 and 7). Scale bar: 50 μm. **(B)** (i–iv), GFAP positive cell area was significantly increased in all the regions (corpus callosum, ipsilateral internal capsule, ipsilateral external capsule, and contralateral external capsule) on day 7 post-injury in pR5-TBI group versus the pR5-sham group, whereas no significant differences were observed the day 1 pR5-TBI group versus the pR5 sham group except ipsilateral internal capsule (**B** (iii)) (one-way ANOVA with Tukey’s multiple comparison test, *p ≤ 0.05, **p ≤ 0.001, ***p ≤ 0.0001). **(C)** (i–iv), Anisotropy was significantly reduced with gliosis in the corpus callosum and ipsilateral internal capsule. Axial diffusion (AxD) was also measurably reduced in the corpus callosum, ipsilateral internal capsule, and contralateral external capsule whereas in the ipsilateral external capsule, axial diffusivity (AxD) was increased significantly with gliosis. Radial and mean diffusivity were also increased in the ipsilateral external capsule with gliosis, whereas, in the contralateral external capsule, mean diffusivity (MD) was reduced with gliosis (Pearson multiple correlations *p*-value ≤ 0.05).

**FIGURE 7:**
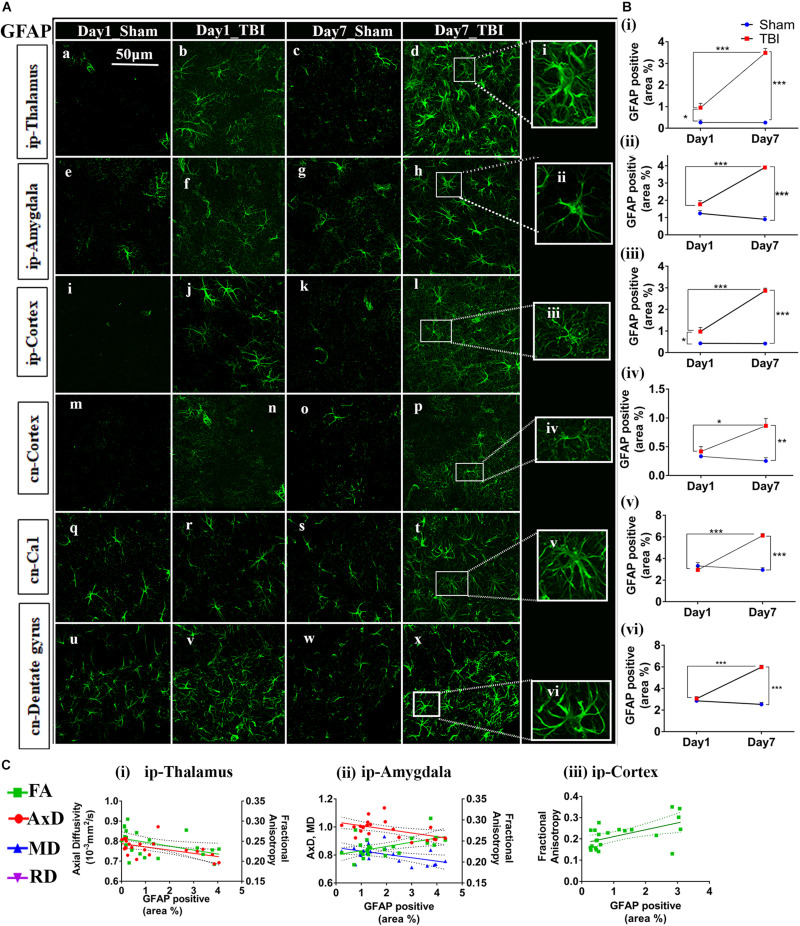
Augmented astrogliosis post-TBI in the gray matter of injured pR5 mice: **(A)** Statistically significant increase in glial fibrillary acidic protein (GFAP)-positive cell area percentage in the gray matter regions, including the ipsilateral thalamus (a–d), ipsilateral amygdala (e–h), ipsilateral cortex (i–k), contralateral cortex (m–p), contralateral CA1 (q–t), and contralateral dentate gyrus (u–x) of pR5 TBI groups versus sham groups (*n* = 6 pR5 TBI mice and *n* = 6 pR5-sham mice at days 1 and 7). Scale bar: 50 μm. Extended views of astrocytes from all regions were shown (i–viii) indicating the differences in the shape of the astrocytes. **(B)** (i–vi), GFAP-positive percentage cell area was significantly higher in the ipsilateral thalamus and ipsilateral cortex on day 1 post-injury in pR5-TBI mice versus the pR5-sham mice control group. On day 7, the percent positive area was evidently increased in ipsilateral thalamus, ipsilateral amygdala, ipsilateral cortex, contralateral cortex, contralateral CA1, and contralateral dentate gyrus (One-way ANOVA with Tukey’s multiple comparison test, *p ≤ 0.05, **p ≤ 0.001, ***p ≤ 0.0001). **(C)** (i–iii), Anisotropy was significantly reduced in the ipsilateral thalamus and increased in the ipsilateral amygdala with gliosis. Axial diffusion was also measurably reduced in the ipsilateral thalamus and ipsilateral amygdala. Reduced mean diffusivity was also noted in the ipsilateral amygdala with increased gliosis (Pearson multiple correlations *p*-value ≤ 0.05).

Overall, increased GFAP expression was evident one day post-TBI that peaked after 7 days in the TBI groups as compared to the sham group (Figures 6A, 7A). In the case of astrocytes, changes were noted in both the percentage of GFAP-positive cell areas (reported below) and GFAP-positive cell count (outlined in Supplementary Table 1). DTI measures-FA, AxD, MD, and RD-showed significant changes with the increased expression of GFAP in several regions of the brain.

In the white matter (Figure 6B), on day 1, a significant increase percentage of GFAP-positive cell area was observed in the ip-internal capsule in the TBI group (3.909 ± 0.5325; *p* = 0.0075) versus the sham group (1.611 ± 0.3459). No significant increase in percentage of GFAP positive cell area was seen in the cn-internal capsule (2.560 ± 0.3460; *p* = 0.0693), and corpus callosum (2.714 ± 0.3160; *p* = 0.2327) when compared to the day 1 sham group; cn-internal capsule (1.427 ± 0.1885), corpus callosum (1.640 ± 0.2749). On day 7, percentage of GFAP-positive staining was significantly higher in the TBI groups in all selected white matter regions, including ip-internal capsule (4.679 ± 0.5405, *p* = 0.0149), corpus callosum (6.165 ± 0.4585; *p* = 0.0001), ip-external capsule (7.089 ± 1.014, *p* = 0.0027), and cn-external capsule (7.570 ± 0.4892, *p* = 0.0003) versus sham regions; ip-internal capsule (2.576 ± 0.2912), corpus callosum (1.893 ± 0.4579), ip-external capsule (3.364 ± 1.156), and cn-external capsule (3.670 ± 0.6898). These findings show that GFAP immunostaining in the white matter increase within a week of injury in the pR5 mice.

In correlating with the DTI measures (Figure 6C), a negative correlation was seen between the percentage of GFAP positive cell area and FA in the corpus callosum (*R*^2^ = 0.3989; *p* = 0.0012), an ip-internal capsule (*R*^2^ = 0.3075; *p* = 0.0049), and with AxD in the corpus callosum (*R*^2^ = 0.3140; *p* = 0.0054), ip-internal capsule (*R*^2^ = 0.3920; *p* = 0.0011), cn-internal capsule (*R*^2^ = 0.4124; *p* = 0.0010), and cn-external capsule (*R*^2^ = 0.3734; *p* = 0.0019). A positive correlation was observed with AxD (*R*^2^ = 0.2210; *p* = 0.0236), MD (*R*^2^ = 0.3083; *p* = 0.0060), and RD (*R*^2^ = 0.3344; *p* = 0.0038) in the ip-external capsule. Overall, this indicates that a decrease in anisotropy and an increase in diffusion in the white matter were associated with an increase in astrocytes.

In the gray matter (Figure 7B), a significant increase in GFAP expression was seen only in the ip-thalamus (0.9537 ± 0.1991, *p* = 0.0300) and ip-cortex (0.9743 ± 0.1880, *p* = 0.0160) on day 1 in the TBI versus the sham group [ip-thalamus (0.2697 ± 0.1126) and ip-cortex (0.4305 ± 0.0474)], with a significant increase in the ip-thalamus (3.486 ± 0.2074, *p* = 0.0001), ip-cortex (2.867 ± 0.1161, *p* = 0.0001), cn-cortex (0.8618 ± 0.1270; *p* = 0.0004), ip-amygdala (3.900 ± 0.0990, *p* = 0.0001), cn-CA1 (6.119 ± 0.1884, *p* = 0.0001), and cn-dentate gyrus (5.619 ± 1.554, *p* = 0.0318) being observed at 7 days post-injury as compared to shams [ip-thalamus (0.2617 ± 0.0755), ip-cortex (0.4200 ± 0.0463), cn-cortex (0.2523 ± 0.0579), ip-amygdala (0.9060 ± 0.1575), cn-CA1 (2.944 ± 0.2041), and cn-dentate gyrus (1.533 ± 0.4619)]. These results suggest that GFAP immunostaining in gray matter was increased within a week in pR5 mice post-injury.

A negative correlation (Figure 7C) in the ip-thalamus (*R*^2^ = 0.2048; *p* = 0.0301) and a positive correlation in the ip-cortex (*R*^2^ = 0.3148; *p* = 0.0053) and ip-amygdala (*R*^2^ = 0.2759; *p* = 0.0101) were observed with FA. A negative correlation was also seen with AxD in the ip-thalamus (*R*^2^ = 0.2837; *p* = 0.0089) and ip-amygdala (*R*^2^ = 0.2682; *p* = 0.0114). In the ip-amygdala, GFAP expression also negatively correlated with MD (*R*^2^ = 0.2467; *p* = 0.0159). These findings demonstrate region-dependent increases and decreases in FA in association with an increase in GFAP immunostaining. Together with the phosphorylated-tau results, the increase in FA was associated with increased GFAP immunostaining with no tauopathy, and the FA decrease was associated with increased GFAP immunostaining with tauopathy.

### Increased Microglial Cell Number 7 Days Post-injury

**FIGURE 8:**
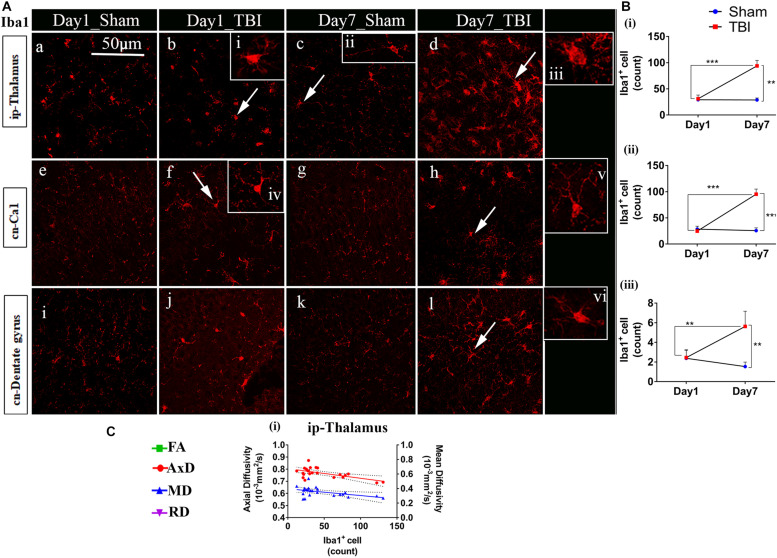
Increased microglia activity in gray matter post-TBI in pR5 mice: **(A)** Iba1+ cell counts were evident in gray matter regions, ipsilateral thalamus (a–d), contralateral CA1 hippocampal region (e–h), and contralateral dentate gyrus (i–l). Extended iba1+ cells (i–vi) demonstrate a difference between active and inactive microglia cells at days 1 and 7 (*n* = 6 pR5 TBI mice and *n* = 6 pR5-sham mice for days 1 and 7). Scale bar: 50 μm. **(B)** (i–iii), A significant increase in microglia count in the ip-thalamus, contralateral CA1, and contralateral dentate gyrus were noticed at day 7 post-injury (One-way ANOVA with Tukey’s multiple comparison test, *p ≤ 0.05, **p ≤ 0.001, ***p ≤ 0.0001). **(C)** Significant negative correlations are observed between Iba1+ cell counts and DTI measures fractional anisotropy (FA), and axial diffusivity (AxD) ipsilateral thalamus (**C** (i)) (Pearson multiple correlations *p*-value ≤ 0.05).

**FIGURE 9:**
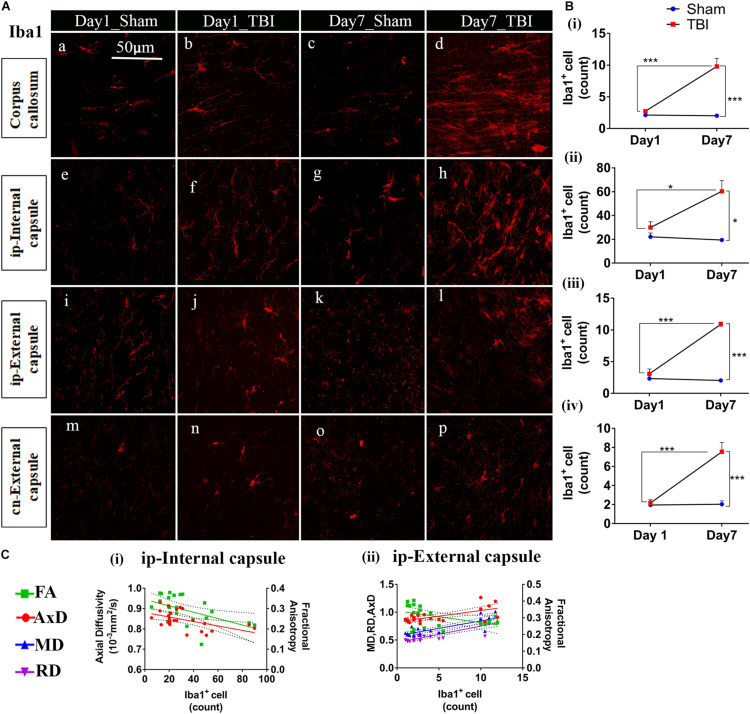
TBI induces microglial increase in the white matter of pR5 mice: **(A)** Increased Iba1+ cell count in white matter regions, including the corpus callosum (a–d), ipsilateral internal capsule (e–h), ipsilateral external capsule (i–l), and contralateral external capsule (m–p), (*n* = 6 pR5 TBI mice and *n* = 6 pR5-sham mice for days 1 and 7-time points). Scale bar: 50 μm. **(B)** (i–iv), Iba1+ cells were significantly higher in day 7 pR5 TBI mice versus sham mice in all four regions (One-way ANOVA with Tukey’s multiple comparison test, *p ≤ 0.05, ** *p* ≤ 0.001, ***p ≤ 0.0001). **(C)** (i,ii), Fractional anisotropy decreased with increased microglia count in the ipsilateral internal capsule and ipsilateral external capsule. Axial diffusivity was decreased in the ipsilateral internal capsule while increased in the ipsilateral external capsule with an increase in microglia count. Along with this, mean and radial diffusivity were also increased with microglia level in the ipsilateral external capsule (Pearson multiple correlations *p*-value ≤ 0.05).

Unlike astrocytes, we did not observe any increase in microglial number (Iba1+ cell count) on day 1; however, active microglial cells were seen in the thalamus region (Figure 8A). A significant increase in microglial cell number was noted only 7 days after injury in both the white matter (Figures 9A,B) corpus callosum (9.796 ± 1.259, *p* = 0.0001), ip-external capsule (10.88 ± 0.3521, *p* = 0.0001), ip-internal capsule (60.33 ± 9.124, *p* = 0.0002), and the gray matter (Figures 8A,B) in the TBI group (ip-thalamus (93.83 ± 10.54; *p* = 0.0001) versus the sham groups–corpus callosum (2.019 ± 0.1608), ip-external capsule (2.042 ± 0.2534), ip-internal capsule (19.42 ± 1.546), and ip-thalamus (28.75 ± 3.857). Notably, in the contralateral regions, a significant increase was observed in the cn-external capsule (7.542 ± 0.9820, *p* = 0.0001), CA1 (95.17 ± 9.862, *p* = 0.0001), and dentate gyrus (73.78 ± 5.007, *p* = 0.0003) at day 7 in the TBI group versus the sham group; cn-external capsule (2.042 ± 0.3501), Ca1 (25.67 ± 5.354), and dentate gyrus (32.39 ± 6.159) regions. These results indicate that microglial activity increases within a week of TBI in pR5 mice.

Although correlated with DTI (Figures 8C, 9C), microglial counts negatively correlated with FA in the ip-external capsule (*R*^2^ = 0.2010; *p* = 0.0319), ip-internal capsule (*R*^2^ = 0.2581; *p* = 0.0113), and with AxD in the ip-internal capsule (*R*^2^ = 0.2755; *p* = 0.0085) and ip-thalamus (*R*^2^ = 0.3631; *p* = 0.0023). A positive correlation was observed with AxD (*R*^2^ = 0.4300; *p* = 0.0007), MD (*R*^2^ = 0.6658; *p* = 0.0001), and RD (*R*^2^ = 0.7362; *p* = 0.0001) in ip-external capsule and negative correlation with MD (*R*^2^ = 0.2143; *p* = 0.0261) in the ip-thalamus. Together, these findings reveal that the white matter anisotropy was mainly affected by the increase in microglial activity whereas in the gray matter diffusivity was primarily affected.

## Discussion

This work revealed evolving and progressive microstructural alterations in tau animals as early as 1 day post-injury that propagated to the contralateral hemisphere within a week, and were associated with TBI-accelerated tauopathy. In the white matter, the reduction in FA and AxD and an increase in MD and RD at day 1 were followed by a decrease in FA, AxD, and MD, and increased RD at seven days post-injury in the TBI-pR5 group compared to shams. In the gray matter, FA was markedly increased in the amygdala and cortex, but remained significantly decreased in the thalamic and hippocampal regions for 1 week as compared to the sham group. The reduced FA was consistently associated with increased expression of phosphorylated tau evident after day 1 in the white matter followed by its propagation to the gray matter within 1 week, in line with neuroinflammation. An increased FA on the other hand, particularly reflected a prominent increase in neuroinflammation without any significant increase in tau phosphorylation. Overall, we observed regional changes in the DTI signal, particularly in FA, that could reflect either increased phosphorylation of tau, neuroinflammation, or both.

Tauopathy has been documented as a possible linking factor between TBI and AD (Johnson et al., 2012). Pathological isoforms of tau have been detected immunohistochemically and biochemically in postmortem TBI brain at various different time points and are shown to affect the behavioral function associated with respective brain areas (Zanier et al., 2018; Zhang et al., 2019). *In vivo* detection of tauopathy at an early stage post-TBI is underexplored. The findings presented here advance our current understanding of TBI-accelerated tauopathy in experimental models by providing information about the early pathological and microstructural changes in a tau transgenic mouse model post single severe injury. This work exploits DTI along with histology to measure widespread pathology in pR5 mice for a period of 1-week post-TBI. This is the first study to apply DTI to investigate the effect of TBI in tau transgenic animals.

In the past few years, tauopathies following TBI have been the hypothesized link with AD (McKee and Daneshvar, 2015). In clinicopathological studies, postmortem TBI brains analyzed at different time points exhibited extensive tau phosphorylation that was associated with cognitive deficits in the patients (Zanier et al., 2018; Zhang et al., 2019). Consistent with previous studies (Acosta et al., 2017; Zhao et al., 2017), we document immunohistologically, a similar increase in tau phosphorylation following TBI. Hyperphosphorylation appeared at day 1 post-injury in the ipsilateral internal capsule then spread to the gray matter regions, specifically to the ipsilateral and contralateral thalamus and the contralateral dentate gyrus after 1 week. Such early changes in tau phosphorylation were consistent with another study that utilized the CCI model in a triple-transgenic mouse (3×Tg-AD), where an increase in phosphorylated-tau expression in the fimbria and the CA1 region of the hippocampus, along with the deposition of amyloid-β plaques within 1 week of the injury were reported (Tran et al., 2011a). Unlike Tran et al., we did not see any increase in total tau levels, which was consistent with the report of Zhang et al. (2019) who reported no changes in total tau levels even 6 weeks after injury. In pR5 mice, NFT formation is initiated at around 6 months in the amygdala and also detectable in the CA1 region at 20 months of age (Deters et al., 2008). In general, the literature supports that aging accelerates the course of AD pathogenesis. Tau hyperphosphorylation was evident in the hippocampus of pR5 mice at 9 months of age post-TBI in our study as compared the typical 20 months of age as reported by previous studies (Gotz et al., 2001; Deters et al., 2008).

The role of neuroinflammation in tau pathogenesis has also been extensively documented (Curran and O’Connor, 2001; Sutinen et al., 2012; Morales et al., 2013; Collins-Praino and Corrigan, 2017; Leyns and Holtzman, 2017; Laurent et al., 2018). In both clinical and experimental models, TBI can augment tau pathology if it coexists with active glial cells (Johnson et al., 2012, 2013; Ojo et al., 2013; Nilson et al., 2017). We noted the presence of reactive astrocytes and activated microglia in regions presenting with increased hyper-phosphorylation, such as the ipsilateral thalamus and contralateral dentate gyrus, corpus callosum, and internal and external capsule. Both microglia and astrocytes have been shown to be promoters for tau hyper-phosphorylation by either increasing the release of pro-inflammatory mediators in their active states that further increases the activity of kinases responsible for tau phosphorylation or by altering other involved pathways (Curran and O’Connor, 2001; Sutinen et al., 2012).

Diffusion tensor imaging is a well-established imaging tool that has been known to detect TBI-induced microstructural abnormalities in the brain (Alexander et al., 2007; Lo et al., 2009; Budde et al., 2011; Soni et al., 2018). Here we have shown, FA and AxD were reduced in the TBI group in the ip-thalamus, corpus callosum, external capsule, and internal capsule compared to the sham transgenic mice with immunohistochemical evidence of increases in tau phosphorylation in these same regions. These findings were consistent with Sahara et al. (2014) where DTI changes in rTg4510 tau transgenic mice were studied with respect to tau pathology, regardless of injury, a similar reduction in FA in the white matter was associated with tau hyper-phosphorylation in rTg4510 mice. In 3×Tg mice, decreased FA was reported primarily in the hippocampal region which was associated with depositions of both Aβ plaques and hyperphosphorylated tau (Snow et al., 2017). Here we noticed tau phosphorylation and reduced FA in the dentate gyrus; however, the correlation was not significant, most likely related to the sensitivity limitations of DTI (Taoka et al., 2009). Another reason could be that the pR5 mouse model used in this study is a tau transgenic mouse with no amyloid pathology; therefore, unlike 3×Tg mice, the changes we noticed were only associated with hyperphosphorylated tau. It should be noted that contradictory to our findings, Wells et al. (2015) reported an opposite trend of FA change in the areas of high tau burden, including the cortex and hippocampus but not in the thalamus where NFT density was significantly low. We also noticed increased FA in a few regions involving the cortical areas close to the injury site–amygdala, ipsilateral epithalamic, and contralateral-ventral thalamic region–but did not find any correlation with phosphorylated-tau increase. It is possible that phosphorylated tau in its aggregated form alters the directionality and diffusion of water in a different manner; however, as we did not look at NFTs, this cannot be confirmed with the current data. Altogether, our results indicate that the post-TBI increase in phosphorylated-tau may induce structural impairments that are measurable with DTI.

The DTI changes observed may be further complicated by the associated neuroinflammatory processes. Astrocytes, that are responsible for upholding homeostasis in the central nervous system (Sofroniew and Vinters, 2010). They are typically been shown to have star like shape and isotropic morphology (Schiweck et al., 2018). However, advanced visualization techniques revealed that shape of astrocytes is complex and keeps changing depending on the region and physiology of the brain (Fernaud-Espinosa et al., 1993). In pathological conditions, such as brain insults, gliomas and neurodegenerative disorders astrocytes appear in different structural forms depending on the severity or stages of the pathological condition (Schiweck et al., 2018). This can either highlight its isotropic behavior or shift it toward anisotropy and can affect directionality of water diffusion. In TBI, some reports have demonstrated isotropic gliosis in gray matter, where astrocytes maintain their distinct shapes but become hypertrophic that may lead to visible decrease in FA (Edlow et al., 2016). Contradictory to these findings, some others have depicted anisomorphic gliosis following injury (Budde et al., 2011; Laitinen et al., 2015; Soni et al., 2018). These studies indicated that astrocytes present in close proximity to injury site have the characteristics to change their shapes by expanding their busy processes toward lesion that lead to anisotropic structure causing increase in FA (Budde et al., 2011; Laitinen et al., 2015; Soni et al., 2018). These astrocytes named as palisading astrocytes are also responsible for the formation of glial scars. In AD, along with morphological variations astrocytes are also demonstrated to have spatial association with the pathological tau which may contribute in disease progression concurrently (Sidoryk-Wegrzynowicz et al., 2017; Kovacs et al., 2018).

In this study, on day 1 post-injury, we observed increase in astrocytes but we did not observe any increase in tau and microglia count. Therefore, slight reduction in FA seen on day 1 can either be because of increased astrocytes, or damage to the fiber bundle attached to the thalamic region or edema. However, as we did not see any significant changes in AxD, RD, and MD thalamic region and also no change in ad was noted in white matter tracks, it is likely indicated toward glial pathology. Generally, microglia activation starts earlier than astrocytes post-TBI but because gliosis had already been reported at 9 months of age in Pr5 animals, it might be possible that TBI induction in these animals resulted in increased astrocyte expression prior to microglial activation seen on days 1 and 7 (Skripuletz et al., 2013; Jha et al., 2019). In our previous TBI study on wild type mouse, we observed an increased FA in the ipsilateral thalamus post 7 days of injury, which was reflecting the anisotropic gliosis (Soni et al., 2018). Whereas in our current study, we observed FA increase only in the cortical area very close to the injury site which can be because of palisading astrocytes and amygdala may reflect neurodegeneration supported by reduction in AD and MD. In thalamus, FA changes were supporting isotropic nature of astrocytes leading to reduction in FA on day 7 post-injury. These differences in FA can be driven by a combined effect of astrocytes, phosphorylated tau and active microglia also seen in the thalamic region on day 7 post-injury. While the association of DTI parameters with neuroinflammatory markers in both gray matter and white matter regions has been extensively studied post-TBI (Budde et al., 2011; Bennett et al., 2012; Soni et al., 2018), the alterations induced by the combined effect of neuroinflammatory markers along with tauopathy has not been explored. In our study, we observed a negative correlation of GFAP expression with FA and AxD change in the areas with phosphorylated-tau increases. This FA reduction with respect to gliosis was opposite to other CCI studies on wild-type rodents, where increased FA was shown to reflect gliosis (Budde et al., 2011; Xu et al., 2011; Soni et al., 2018). This inconsistent pattern could be explained by the additional presence of phosphorylated-tau and microglia in the thalamus of transgenic mice post-injury, that might be absent in those wild-type rodents. Thus, it could be suggested that these changes are either driven by both the astrocytes or phosphorylated tau together or dominated by phosphorylated tau (Sahara et al., 2014; Snow et al., 2017). In contrast to the thalamus, in the ip-amygdala and cortex, we observed a positive correlation between astrocytes and FA, which was consistent with the TBI studies (Budde et al., 2011; Soni et al., 2018). Thus, it justifies that the changes were only driven by astrocytes as the phosphorylated-tau burden in these areas did not differ between sham and TBI groups. The negative correlation of microglia counts with MD and AxD in the thalamus suggested that the increase in the microglial cell density in the thalamic region could restrict water diffusion in the area. However, as TBI initiated multifaceted mechanisms and DTI measures might be affected by a multitude of factors other than phosphorylation and neuroinflammatory processes, investigation of other processes would contribute further to the specificity of these effects. Other pathological mechanisms, such as axonal injury, amyloid deposition (Tran et al., 2011b; Genrikhs et al., 2017), neuronal death (Murakami et al., 1998; Genrikhs et al., 2017), neurogenesis (Tran et al., 2006), and demyelination (Guglielmetti et al., 2016) may also contribute to the changes observed in DTI measures specifically FA (Rola et al., 2006). Axonal injury to the axons may perturb the dissociation of tau protein from the microtubules leading to microtubular destabilization and increased tau protein levels in the brain tissue, cerebrospinal fluid, and serum/plasma (Zemlan et al., 1999; Smith et al., 2003; Johnson et al., 2013). When evaluated in TBI patients and experimental models it has been found that both total tau and phosphorylated tau levels were significantly increased and were affecting memory functions (Zhao et al., 2017; Zanier et al., 2018). These findings suggested that axonal injury is not the only mechanism responsible for TBI associated tauopathy. Axonal damage dissociates tau protein from the microtubule and may provide a surge to other underlying pathological mechanisms that can further accelerate and worsen the tau pathology by initiating tau hyperphosphorylation and oligomer formation. Axonal injury together with inflammation, cell death and myelin damage can affect diffusion differently at one and 7 days after the injury. Therefore, investigating transgenic models, where key processes are more prominent and elevated than others, may be a key approach to highlighting the DTI alterations that may be more reflective of those processes.

Our study has several limitations, which if addressed, could aid in a better understanding of the associated pathological process. This study shows that TBI affects increasing tau pathology in the pR5 mice, it is unable to conclude whether TBI has more of an effect in the presence of tau pathology since no comparison was made in normal mice. Therefore, the inclusion of non-transgenic sham and TBI littermates would have provided a suitable baseline for comparisons. Further, behavioral assessments of cognition were not performed. The lack of cognitive testing in this study means that this study cannot define the functional impact of the DTI and pathological changes that we have detected, and this warrants future study. The investigation at longer follow-up times would provide insight into the trajectory of tau propagation in pR5 mice and allow the development of better diagnosis paradigm. Although DTI changes were observed in the regions with a high burden of phospho-tau, astrocytes and microglia due to its nature of detecting complex pathologies, it cannot be said that the changes observed in DTI measures are specifically due to these pathological events as the direct correlation were not performed. The limitation on direct matching of DTI and histological measurements are inherent to the methods themselves: MRI can image the whole brain while in general, histology can only image a few very thin sections per animal. Even a single MRI slice is approximately 10 times thicker than one histological slice. Nevertheless, the specific correlation of a histopathological process with a specific trend of diffusion imaging metric change is a complicated issue. For example, a specific pathology may result in a predictable biomarker on DTI or NODDI; however, different pathologies may create the same diffusion metric change (Jelescu and Budde, 2017). Furthermore, in complex conditions like concussion or TBI, pathologies generally do not occur alone. Astrogliosis, microgliosis, and axonal injury generally occur together as a consequence of TBI in mice (Namjoshi et al., 2016, 2017; Bashir et al., 2020; To et al., 2020). Thus, we placed greater importance on maintaining consistency within the same modality (MRI or histology) across different subjects rather than across different modalities (MRI and histology) within the same subject. For consistency across all subjects with MRI, the same ROI covering the defined by the AMBMC atlas was used. For histology, we used the same box ROI size for different subjects covering approximately the same area. Greater importance was placed on determining the capability of DTI on detecting the effects of injury by comparing the sham and controls and demonstrating the underlying pathologies underpinning in injured animals. The correlation between DTI and histological measurements were serendipitous in nature. Also, Fourier or structure tensor-based analyses (Budde et al., 2011) can provide anisotropy to directly compare to FA. However, as this was the first MRI study in TBI-tau transgenic mice model, our purpose was to first understand the pathological cascade if there is any pathological change at early stage and how it can affect DTI measures for which it was important to perform staining. As from this study it is now clear the microstructural changes involved in this double model are mainly affecting FA. In our proposed future study, we will be specifically performing a correlation study and will be using Fourier or structure tensor-based analyses. Another major limitation of this study was the lack of evaluation of sex differences which indeed can increase our understating of disease progression and spatiotemporal changes with time (Wright et al., 2017a, b). A comprehensive comparison of disease progression is a critical step which is required to implement the diagnostic and therapeutic tools at a clinical level thus can be suggested to include in the future studies. Further, a sample size of *n* = 6 per group was used for this study. to calculate the sample size of this study we conducted a power analysis based on our previously published work; we obtained a standard deviation obtained in the DTI measures (FA and mean diffusivity) from first study, which was 20% relative to the mean, six mice per surgery group were required to be able to observe a 22% difference with alpha of 0.05 and 80% power. Further, with the standard deviation found in immunohistochemistry in the pilot experiments (which was 25% relative to the mean), we needed six mice per surgery group to be able to observe 27% of differences at the 0.05 significance level and 80% power. However, considering the heterogeneous of the model and pathologies evolved post-injury in CCI, we suggest the use of large data sets for similar future studies.

## Conclusion

In conclusion, the detection of the cumulative effects of TBI in the AD brain is limited because of the myriad of ongoing processes. We highlight that DTI, and in particular, FA patterns as promising methods to discern tauopathy-related pathology compared to combined tau and neuroinflammatory processes in the brain. These findings are supported by our histological measures of tau, microglia, and astrogliosis. We suggest FA as a potential *in vivo* marker to detect TBI-associated tauopathy and this would be the first study to demonstrate the spatio-temporal profile of DTI microstructural changes following TBI in a transgenic tau model. Our findings advance the current understanding of diagnostic tools for the detection of TBI-accelerated tauopathy in experimental animal models.

## Data Availability Statement

The raw data supporting the conclusions of this article will be made available by the authors, without undue reservation.

## Ethics Statement

The animal study was reviewed and approved by Animal Research Ethics Committee of the University of Queensland (Animal Ethics Committee number: QBI/SCMB/036/16/MAIC).

## Author Contributions

NS and FN have contributed to design of the study and data interpretation. NS contributed to generate the animal model, data acquisition, analysis, drafting the manuscript. RM revised the manuscript for important intellectual content. XVT contributed to manuscript revision during the review process. KA helped in data acquisition. FN provided approval for publication of the content. All authors contributed to the article and approved the submitted version.

## Conflict of Interest

The authors declare that the research was conducted in the absence of any commercial or financial relationships that could be construed as a potential conflict of interest.

## Footnotes

1 https://fsl.fmrib.ox.ac.uk/fsl/fslwiki

2 http://imaging.org.au/AMBMC/Model

3 https://alleninstitute.org/

## Supplementary Material

Supplementary Figure 1 Example of distortion-corrected data from a sham subject, demonstrating SNR.

Supplementary Figure 2 Examples of registered FA maps from sham and CCI animals of days 2 and 7. Each individual’s registered FA maps (grayscale background) were shown with the corresponding skeletonized FA maps (rainbow scaled overlays). Generally, thinner white matter tracts (e.g., the external capsule on contralateral side) were well-matched across subjects, evidenced by the thin FA skeleton consistently covering the whole tract. Thicker white matter tracts, i.e., areas with high FA, (e.g., the corpus callosum and internal capsule, indicated on first row with purple arrows) had slight mismatches, in terms of the tract thickness and/or the FA skeleton did not always pass through the same location along the tract. TBSS skeletonizing process improve the spatial matching of these white matter tracts and overall, the skeletonizing process was accurate, i.e., the FA skeleton always fall within larger FA tracts. TBSS also reflected situation where the white matter tract was disrupted in lesion area in the CCI animals, for example the ipsilateral external capsule (indicated by light blue arrows). On the registered FA maps, the external capsule in the lesion area disappeared and the corresponding FA skeleton passing through the area, the FA value was significantly lower to than the contralateral external capsule FA skeletonized FA tract (∼0.1, below the TBSS FA threshold). The white matter tracts were grossly aligned and consequently the more homogenous (in terms of DTI metrics pattern) gray matter area was sufficiently well-matched for voxel-wise spatial statistics. This is relevant since TBSS does not provide results on DTI changes in the gray matter.

Supplementary Figure 3 Figure representing the sections and regions of interest chosen for region-based quantification: **(A)** Representative images from the developing Allen mouse brain atlas to demonstrate the bregma region (−0.5 to −4.5 mm) used for immunohistochemistry analysis and quantification. **(B)** Images of the MRI slices from the same bregma region were chosen to demonstrate the Regions-of-interest that were drawn manually on the study-specific template using the Australian mouse brain mapping consortium template (Watson et al., 2017) and the Allen adult mouse brain atlas (https://alleninstitute.org/) as a reference. All slices between the bregma regions −0.5 to −4.5 mm were covered in the ROI mask. **(C)** Representative serial coronal sections chosen for immunohistochemistry quantification. Regions of interest were Amygdala, thalamus, hippocampus (CA1 and dentate gyrus), cortex (closer to the amygdala region), corpus callosum, internal and external capsule shown with different colors on the slices. Two sample boxes (one large and one small) were used to create multiple ROIs for all the slices, that were used to quantify pathologies in different areas as shown in different colors in the figure. To be consistent with the size of the ROIs, same set of ROIs for all the five sections were used for all the animals with slight adjustment in the location if needed.

Supplementary Figure 4 Histology sections of a representative sham mouse stained for p-tau.

## References

[B1] Acosta S. A. Tajiri N. Sanberg P. R. Kaneko Y. Borlongan C. V. (2017). Increased amyloid precursor protein and tau expression manifests as key secondary cell death in chronic traumatic brain injury. *J. Cell. Physiol.* 232 665–677. 10.1002/jcp.25629 27699791 PMC5484295

[B2] Alexander A. L. Lee J. E. Lazar M. Field A. S. (2007). Diffusion tensor imaging of the brain. *Neurotherapeutics* 4 316–329. 17599699 10.1016/j.nurt.2007.05.011 PMC2041910

[B3] Andersson J. L. Skare S. Ashburner J. (2003). How to correct susceptibility distortions in spin-echo echo-planar images: application to diffusion tensor imaging. *Neuroimage* 20 870–888. 10.1016/s1053-8119(03)00336-7 14568458

[B4] Bashir A. Abebe Z. A. McInnes K. A. Button E. B. Tatarnikov I. Cheng W. H. (2020). Increased severity of the CHIMERA model induces acute vascular injury, sub-acute deficits in memory recall, and chronic white matter gliosis. *Exp. Neurol.* 324:113116. 10.1016/j.expneurol.2019.113116 31734317

[B5] Beaulieu C. (2002). The basis of anisotropic water diffusion in the nervous system - a technical review. *NMR Biomed.* 15 435–455. 10.1002/nbm.782 12489094

[B6] Behrens T. E. J. Woolrich M. W. Jenkinson M. Johansen-Berg H. Nunes R. G. Clare S. (2003). Characterization and propagation of uncertainty in diffusion-weighted MR imaging. *Magn. Reson. Med.* 50 1077–1088. 14587019 10.1002/mrm.10609

[B7] Bemiller S. M. Mccray T. J. Allan K. Formica S. V. Xu G. X. Wilson G. (2017). TREM2 deficiency exacerbates tau pathology through dysregulated kinase signaling in a mouse model of tauopathy. *Mol. Neurodegenerat.* 12:74. 10.1186/s13024-017-0216-6 PMC5644120 29037207

[B8] Bennett R. E. Mac Donald C. L. Brody D. L. (2012). Diffusion tensor imaging detects axonal injury in a mouse model of repetitive closed-skull traumatic brain injury. *Neurosci. Lett.* 513 160–165. 10.1016/j.neulet.2012.02.024 22343314 PMC3319388

[B9] Bodea L. G. Evans H. T. Van Der Jeugd A. Ittner L. M. Delerue F. Kril J. (2017). Accelerated aging exacerbates a pre-existing pathology in a tau transgenic mouse model. *Aging Cell* 16 377–386. 10.1111/acel.12565 28160413 PMC5334525

[B10] Buckley R. F. Hanseeuw B. Schultz A. P. Vannini P. Aghjayan S. L. Properzi M. J. (2017). Region-specific association of subjective cognitive decline with tauopathy independent of global beta-amyloid burden. *JAMA Neurol.* 74 1455–1463. 10.1001/jamaneurol.2017.2216 28973551 PMC5774633

[B11] Budde M. D. Janes L. Gold E. Turtzo L. C. Frank J. A. (2011). The contribution of gliosis to diffusion tensor anisotropy and tractography following traumatic brain injury: validation in the rat using Fourier analysis of stained tissue sections. *Brain* 134 2248–2260. 10.1093/brain/awr161 21764818 PMC3155707

[B12] Castellani R. J. Perry G. (2019). Tau biology, tauopathy, traumatic brain injury, and diagnostic challenges. *J. Alzheimers Dis. JAD* 67 447–467. 10.3233/jad-180721 30584140 PMC6398540

[B13] Chou N. Wu J. Bai Bingren J. Qiu A. Chuang K. H. (2011). Robust automatic rodent brain extraction using 3-D pulse-coupled neural networks (PCNN). *IEEE Trans. Image Process* 20 2554–2564. 10.1109/tip.2011.2126587 21411404

[B14] Collins-Praino L. E. Corrigan F. (2017). Does neuroinflammation drive the relationship between tau hyperphosphorylation and dementia development following traumatic brain injury? *Brain Behav. Immun.* 60 369–382. 10.1016/j.bbi.2016.09.027 27686843

[B15] Curran B. O’Connor J. J. (2001). The pro-inflammatory cytokine interleukin-18 impairs long-term potentiation and NMDA receptor-mediated transmission in the rat hippocampus in vitro. *Neuroscience* 108 83–90. 10.1016/s0306-4522(01)00405-5 11738133

[B16] Deters N. Ittner L. M. Gotz J. (2008). Divergent phosphorylation pattern of tau in P301L tau transgenic mice. *Eur. J. Neurosci.* 28 137–147. 10.1111/j.1460-9568.2008.06318.x 18662339

[B17] Edlow B. L. Copen W. A. Izzy S. Bakhadirov K. Van Der Kouwe A. Glenn M. B. (2016). Diffusion tensor imaging in acute-to-subacute traumatic brain injury: a longitudinal analysis. *BMC Neurol.* 16:2. 10.1186/s12883-015-0525-8 26754948 PMC4707723

[B18] Fann J. R. Ribe A. R. Pedersen H. S. Fenger-Gron M. Christensen J. Benros M. E. (2018). Long-term risk of dementia among people with traumatic brain injury in Denmark: a population-based observational cohort study. *Lancet Psychiatry* 5 424–431. 10.1016/s2215-0366(18)30065-8 29653873

[B19] Fernaud-Espinosa I. Nieto-Sampedro M. Bovolenta P. (1993). Differential activation of microglia and astrocytes in aniso- and isomorphic gliotic tissue. *Glia* 8 277–291. 10.1002/glia.440080408 8406684

[B20] Fichou Y. Schiro G. Gallat F. X. Laguri C. Moulin M. Combete J. (2015). Hydration water mobility is enhanced around tau amyloid fibers. *Proc. Natl. Acad. Sci. U.S.A.* 112, 6365–6370. 10.1073/pnas.1422824112 25918405 PMC4443308

[B21] Fleminger S. Oliver D. L. Lovestone S. Rabe-Hesketh S. Giora A. (2003). Head injury as a risk factor for Alzheimer’s disease: the evidence 10 years on; a partial replication. *J. Neurol. Neurosurg. Psychiatry* 74, 857–862. 10.1136/jnnp.74.7.857 12810767 PMC1738550

[B22] Franklin K. Paxinos G. (1997). *The Mouse Brain in Stereotaxic Coordinates.* San Diego, CA: Academic Press.

[B23] Genrikhs E. E. Voronkov D. N. Kapkaeva M. R. Isaev N. K. Stelmashook E. V. (2017). Focal unilateral traumatic brain injury causes delayed neurodegenerative changes in the brain of rats. *Bull. Exp. Biol. Med.* 164 211–213. 10.1007/s10517-017-3960-2 29177900

[B24] Gotz J. Chen F. Barmettler R. Nitsch R. M. (2001). Tau filament formation in transgenic mice expressing P301L tau. *J. Biol. Chem.* 276 529–534. 10.1074/jbc.m006531200 11013246

[B25] Guglielmetti C. Veraart J. Roelant E. Mai Z. Daans J. Van Audekerke J. (2016). Diffusion kurtosis imaging probes cortical alterations and white matter pathology following cuprizone induced demyelination and spontaneous remyelination. *Neuroimage* 125 363–377. 10.1016/j.neuroimage.2015.10.052 26525654 PMC4935929

[B26] Hanger D. P. Anderton B. H. Noble W. (2009). Tau phosphorylation: the therapeutic challenge for neurodegenerative disease. *Trends Mol. Med.* 15 112–119. 10.1016/j.molmed.2009.01.003 19246243

[B27] Hay J. Johnson V. E. Smith D. H. Stewart W. (2016). Chronic traumatic encephalopathy: the neuropathological legacy of traumatic brain injury. *Annu. Rev. Pathol.* 11, 21–45. 10.1146/annurev-pathol-012615-044116 26772317 PMC5367053

[B28] Jelescu I. O. Budde M. D. (2017). Design and validation of diffusion MRI models of white matter. *Front. Phys.* 28:61. 10.3389/fphy.2017.00061 29755979 PMC5947881

[B29] Jha M. K. Jo M. Kim J. H. Suk K. (2019). Microglia-astrocyte crosstalk: an intimate molecular conversation. *Neuroscientist* 25 227–240. 10.1177/1073858418783959 29931997

[B30] Johnson K. A. Schultz A. Betensky R. A. Becker J. A. Sepulcre J. Rentz D. (2016). Tau positron emission tomographic imaging in aging and early Alzheimer disease. *Ann. Neurol.* 79 110–119. 10.1002/ana.24546 26505746 PMC4738026

[B31] Johnson V. E. Stewart J. E. Begbie F. D. Trojanowski J. Q. Smith D. H. Stewart W. (2013). Inflammation and white matter degeneration persist for years after a single traumatic brain injury. *Brain* 136 28–42. 10.1093/brain/aws322 23365092 PMC3562078

[B32] Johnson V. E. Stewart W. Smith D. H. (2012). Widespread tau and amyloid-beta pathology many years after a single traumatic brain injury in humans. *Brain Pathol.* 22 142–149. 10.1111/j.1750-3639.2011.00513.x 21714827 PMC3979351

[B33] Kovacs G. G. Xie S. X. Robinson J. L. Lee E. B. Smith D. H. Schuck T. (2018). Sequential stages and distribution patterns of aging-related tau astrogliopathy (ARTAG) in the human brain. *Acta Neuropathol. Commun.* 6:50. 10.1186/s40478-018-0552-y PMC5996526 29891013

[B34] Lagarde J. Sarazin M. Bottlaender M. (2018). In vivo PET imaging of neuroinflammation in Alzheimer’s disease. *J. Neural Transm.* 125 847–867. 10.1007/s00702-017-1731-x 28516240

[B35] Laitinen T. Sierra A. Bolkvadze T. Pitkanen A. Grohn O. (2015). Diffusion tensor imaging detects chronic microstructural changes in white and gray matter after traumatic brain injury in rat. *Front. Neurosci.* 9:128. 10.3389/fnins.2015.00128 25954146 PMC4406060

[B36] Laurent C. Buee L. Blum D. (2018). Tau and neuroinflammation: what impact for Alzheimer’s disease and tauopathies? *Biomed. J.* 41 21–33. 10.1016/j.bj.2018.01.003 29673549 PMC6138617

[B37] Leyns C. E. G. Holtzman D. M. (2017). Glial contributions to neurodegeneration in tauopathies. *Mol. Neurodegener.* 12:50. 10.1186/s13024-017-0192-x PMC5492997 28662669

[B38] Li Y. J. Li Y. M. Li X. T. Zhang S. Zhao J. C. Zhu X. F. (2017). Head Injury as a Risk Factor for Dementia and Alzheimer’s Disease: A Systematic Review and Meta-Analysis of 32 Observational Studies. *PLoS One* 12:e0169650. 10.1371/journal.pone.0169650 28068405 PMC5221805

[B39] Lo C. Shifteh K. Gold T. Bello J. A. Lipton M. L. (2009). Diffusion tensor imaging abnormalities in patients with mild traumatic brain injury and neurocognitive impairment. *J. Comput. Assist. Tomogr.* 33 293–297. 10.1097/rct.0b013e31817579d1 19346863

[B40] Magnoni S. Esparza T. J. Conte V. Carbonara M. Carrabba G. Holtzman D. M. (2012). Tau elevations in the brain extracellular space correlate with reduced amyloid-beta levels and predict adverse clinical outcomes after severe traumatic brain injury. *Brain* 135 1268–1280. 10.1093/brain/awr286 22116192 PMC3326246

[B41] McKee A. C. Daneshvar D. H. (2015). The neuropathology of traumatic brain injury. *Handb. Clin. Neurol.* 127 45–66. 10.1016/b978-0-444-52892-6.00004-0 25702209 PMC4694720

[B42] Morales I. Jimenez J. M. Mancilla M. Maccioni R. B. (2013). Tau oligomers and fibrils induce activation of microglial cells. *J. Alzheimers. Dis.* 37 849–856. 10.3233/jad-131843 23948931

[B43] Murakami N. Yamaki T. Iwamoto Y. Sakakibara T. Kobori N. Fushiki S. (1998). Experimental brain injury induces expression of amyloid precursor protein, which may be related to neuronal loss in the hippocampus. *J. Neurotrauma* 15 993–1003. 10.1089/neu.1998.15.993 9840772

[B44] Namjoshi D. R. Cheng W. H. Bashir A. Wilkinson A. Stukas S. Martens K. M. (2017). Defining the biomechanical and biological threshold of murine mild traumatic brain injury using CHIMERA (closed head impact model of engineered rotational acceleration). *Exp. Neurol.* 292 80–91. 10.1016/j.expneurol.2017.03.003 28274861

[B45] Namjoshi D. R. Cheng W. H. Carr M. Martens K. M. Zareyan S. Wilkinson A. (2016). Chronic exposure to androgenic-anabolic steroids exacerbates axonal injury and microgliosis in the CHIMERA mouse model of repetitive concussion. *PLoS One* 11:e0146540. 10.1371/journal.pone.0146540 26784694 PMC4718534

[B46] Nilson A. N. English K. C. Gerson J. E. Barton Whittle T. Nicolas Crain C. Xue J. (2017). Tau oligomers associate with inflammation in the brain and retina of tauopathy mice and in neurodegenerative diseases. *J. Alzheimers. Dis.* 55 1083–1099. 10.3233/jad-160912 27716675 PMC5147514

[B47] Ojo J. O. Mouzon B. Greenberg M. B. Bachmeier C. Mullan M. Crawford F. (2013). Repetitive mild traumatic brain injury augments tau pathology and glial activation in aged hTau mice. *J. Neuropathol. Exp. Neurol.* 72 137–151. 10.1097/nen.0b013e3182814cdf 23334597

[B48] Pischiutta F. Micotti E. Hay J. R. Marongiu I. Sammali E. Tolomeo D. (2018). Single severe traumatic brain injury produces progressive pathology with ongoing contralateral white matter damage one year after injury. *Exp. Neurol.* 300 167–178. 10.1016/j.expneurol.2017.11.003 29126888 PMC5745280

[B49] Rola R. Mizumatsu S. Otsuka S. Morhardt D. R. Noble-Haeusslein L. J. Fishman K. (2006). Alterations in hippocampal neurogenesis following traumatic brain injury in mice. *Exp. Neurol.* 202 189–199. 10.1016/j.expneurol.2006.05.034 16876159

[B50] Rose S. E. Janke A. L. Chalk J. B. (2008). Gray and white matter changes in Alzheimer’s disease: a diffusion tensor imaging study. *J. Magn. Reson. Imaging* 27 20–26. 10.1002/jmri.21231 18050329

[B51] Rubenstein R. Chang B. Yue J. K. Chiu A. Winkler E. A. Puccio A. M. (2017). Comparing plasma phospho tau, total tau, and phospho tau-total tau ratio as acute and chronic traumatic brain injury biomarkers. *JAMA Neurol.* 74 1063–1072. 10.1001/jamaneurol.2017.0655 28738126 PMC5710183

[B52] Sahara N. Perez P. D. Lin W. L. Dickson D. W. Ren Y. Zeng H. (2014). Age-related decline in white matter integrity in a mouse model of tauopathy: an in vivo diffusion tensor magnetic resonance imaging study. *Neurobiol. Aging* 35 1364–1374. 10.1016/j.neurobiolaging.2013.12.009 24411290 PMC4729397

[B53] Schiweck J. Eickholt B. J. Murk K. (2018). Important shapeshifter: mechanisms allowing astrocytes to respond to the changing nervous system during development, injury and disease. *Front. Cell. Neurosci.* 12:261. 10.3389/fncel.2018.00261 30186118 PMC6111612

[B54] Sidoryk-Wegrzynowicz M. Gerber Y. N. Ries M. Sastre M. Tolkovsky A. M. Spillantini M. G. (2017). Astrocytes in mouse models of tauopathies acquire early deficits and lose neurosupportive functions. *Acta Neuropathol. Commun.* 5:89. 10.1186/s40478-017-0478-9 PMC6389177 29187256

[B55] Skripuletz T. Hackstette D. Bauer K. Gudi V. Pul R. Voss E. (2013). Astrocytes regulate myelin clearance through recruitment of microglia during cuprizone-induced demyelination. *Brain* 136 147–167. 10.1093/brain/aws262 23266461

[B56] Smith C. Graham D. I. Murray L. S. Nicoll J. A. (2003). Tau immunohistochemistry in acute brain injury. *Neuropathol. Appl. Neurobiol.* 29, 496–502. 10.1046/j.1365-2990.2003.00488.x 14507341

[B57] Smith S. M. Jenkinson M. Johansen-Berg H. Rueckert D. Nichols T. E. Mackay C. E. (2006). Tract-based spatial statistics: voxelwise analysis of multi-subject diffusion data. *Neuroimage* 31 1487–1505. 10.1016/j.neuroimage.2006.02.024 16624579

[B58] Smith S. M. Jenkinson M. Woolrich M. W. Beckmann C. F. Behrens T. E. Johansen-Berg H. (2004). Advances in functional and structural MR image analysis and implementation as FSL. *Neuroimage* 23(Suppl. 1), S208–S219. 15501092 10.1016/j.neuroimage.2004.07.051

[B59] Smith S. M. Johansen-Berg H. Jenkinson M. Rueckert D. Nichols T. E. Miller K. L. (2007). Acquisition and voxelwise analysis of multi-subject diffusion data with tract-based spatial statistics. *Nat. Protoc.* 2 499–503. 10.1038/nprot.2007.45 17406613

[B60] Snow W. M. Dale R. O’brien-Moran Z. Buist R. Peirson D. Martin M. (2017). In vivo detection of gray matter neuropathology in the 3xTg mouse model of Alzheimer’s disease with diffusion tensor imaging. *J. Alzheimers Dis.* 58 841–853. 10.3233/jad-170136 28505976 PMC5467717

[B61] Sofroniew M. V. Vinters H. V. (2010). Astrocytes: biology and pathology. *Acta Neuropathol.* 119 7–35. 10.1007/s00401-009-0619-8 20012068 PMC2799634

[B62] Soni N. Mohamed A. Z. Kurniawan N. D. Borges K. Nasrallah F. (2018). Diffusion MRI unveils the spatiotemporal microstructural grey matter changes following injury in the rodent brain. *J. Neurotrauma.* 36 1306–1317. 10.1089/neu.2018.5972 30381993

[B63] Soni N. Vegh V. To X. V. Mohamed A. Z. Borges K. Nasrallah F. A. (2020). Combined diffusion tensor imaging and quantitative susceptibility mapping discern discrete facets of white matter pathology post-injury in the rodent brain. *Front. Neurol.* 11:153. 10.3389/fneur.2020.00153 32210907 PMC7067826

[B64] Sutinen E. M. Pirttila T. Anderson G. Salminen A. Ojala J. O. (2012). Pro-inflammatory interleukin-18 increases Alzheimer’s disease-associated amyloid-beta production in human neuron-like cells. *J. Neuroinflammation* 9:199. 10.1186/1742-2094-9-199 PMC3458954 22898493

[B65] Taoka T. Morikawa M. Akashi T. Miyasaka T. Nakagawa H. Kiuchi K. (2009). Fractional anisotropy-threshold dependence in tract-based diffusion tensor analysis: evaluation of the uncinate fasciculus in Alzheimer disease. *Am. J. Neuroradiol.* 30 1700–1703. 19541775 10.3174/ajnr.A1698 PMC7051508

[B66] To X. V. Benetatos J. Soni N. Liu D. Abraha H. M. Yan W. (2020). Ultra-high-field diffusion tensor imaging identifies discrete patterns of concussive injury in the rodent brain. *J. Neurotrauma.* [Epub ahead of print]. 10.1089/neu.2019.6944 32394788

[B67] Tran H. T. Laferla F. M. Holtzman D. M. Brody D. L. (2011a). Controlled cortical impact traumatic brain injury in 3xTg-AD mice causes acute intra-axonal amyloid-beta accumulation and independently accelerates the development of tau abnormalities. *J. Neurosci.* 31 9513–9525. 10.1523/jneurosci.0858-11.2011 21715616 PMC3146343

[B68] Tran H. T. Sanchez L. Esparza T. J. Brody D. L. (2011b). Distinct temporal and anatomical distributions of amyloid-beta and tau abnormalities following controlled cortical impact in transgenic mice. *PLoS One* 6:e25475. 10.1371/journal.pone.0025475 21980472 PMC3183029

[B69] Tran L. D. Lifshitz J. Witgen B. M. Schwarzbach E. Cohen A. S. Grady M. S. (2006). Response of the contralateral hippocampus to lateral fluid percussion brain injury. *J. Neurotrauma* 23 1330–1342. 10.1089/neu.2006.23.1330 16958585

[B70] Wang Q. Wang Y. Shimony J. S. Fagan A. M. Cairns N. J. Ances B. (2015). Diffusion tensor imaging detected neurodegeneration in preclinical Alzheimer disease. *Alzheimers Dement. J. Alzheimers Assoc.* 11 77–78.

[B71] Watson C. Janke A. L. Hamalainen C. Bagheri S. M. Paxinos G. Reutens D. C. (2017). An ontologically consistent MRI-based atlas of the mouse diencephalon. *Neuroimage* 157 275–287. 10.1016/j.neuroimage.2017.05.057 28578128

[B72] Wells J. A. O’callaghan J. M. Holmes H. E. Powell N. M. Johnson R. A. Siow B. (2015). In vivo imaging of tau pathology using multi-parametric quantitative MRI. *Neuroimage* 111 369–378. 10.1016/j.neuroimage.2015.02.023 25700953 PMC4626540

[B73] Wright D. K. Liu S. Van Der Poel C. Mcdonald S. J. Brady R. D. Taylor L. (2017a). Traumatic brain injury results in cellular, structural and functional changes resembling motor neuron disease. *Cereb. Cortex* 27 4503–4515. 27566977 10.1093/cercor/bhw254

[B74] Wright D. K. O’brien T. J. Shultz S. R. Mychasiuk R. (2017b). Sex matters: repetitive mild traumatic brain injury in adolescent rats. *Ann. Clin. Transl. Neurol.* 4 640–654. 10.1002/acn3.441 28904986 PMC5590540

[B75] Xu S. Zhuo J. Racz J. Shi D. Roys S. Fiskum G. (2011). Early microstructural and metabolic changes following controlled cortical impact injury in rat: a magnetic resonance imaging and spectroscopy study. *J. Neurotrauma* 28 2091–2102. 10.1089/neu.2010.1739 21761962 PMC3191366

[B76] Yoshiyama Y. Uryu K. Higuchi M. Longhi L. Hoover R. Fujimoto S. (2005). Enhanced neurofibrillary tangle formation, cerebral atrophy, and cognitive deficits induced by repetitive mild brain injury in a transgenic tauopathy mouse model. *J. Neurotrauma* 22 1134–1141. 16238489 10.1089/neu.2005.22.1134

[B77] Zanier E. R. Bertani I. Sammali E. Pischiutta F. Chiaravalloti M. A. Vegliante G. (2018). Induction of a transmissible tau pathology by traumatic brain injury. *Brain* 141 2685–2699. 30084913 10.1093/brain/awy193 PMC6113646

[B78] Zemlan F. P. Rosenberg W. S. Luebbe P. A. Campbell T. A. Dean G. E. Weiner N. E. (1999). Quantification of axonal damage in traumatic brain injury: affinity purification and characterization of cerebrospinal fluid tau proteins. *J. Neurochem.* 72, 741–750. 10.1046/j.1471-4159.1999.0720741.x 9930748

[B79] Zhang Y. Wu F. Iqbal K. Gong C. X. Hu W. Liu F. (2019). Subacute to chronic Alzheimer-like alterations after controlled cortical impact in human tau transgenic mice. *Sci. Rep.* 9:3789. 10.1038/s41598-019-40678-4 PMC6405988 30846870

[B80] Zhao Z. A. Ning Y. L. Li P. Yang N. Peng Y. Xiong R. P. (2017). Widespread hyperphosphorylated tau in the working memory circuit early after cortical impact injury of brain (Original study). *Behav. Brain Res.* 323 146–153. 28163095 10.1016/j.bbr.2017.02.002

