# Supplementary figures and images for: Diffusion Tensor Imaging Detects Acute Pathology-Specific Changes in the P301L Tauopathy Mouse Model Following Traumatic Brain Injury
Source: Front Neurosci. 2021 Feb 24;15:611451. doi: 10.3389/fnins.2021.611451 (PMC7943881; doi:10.3389/fnins.2021.611451)

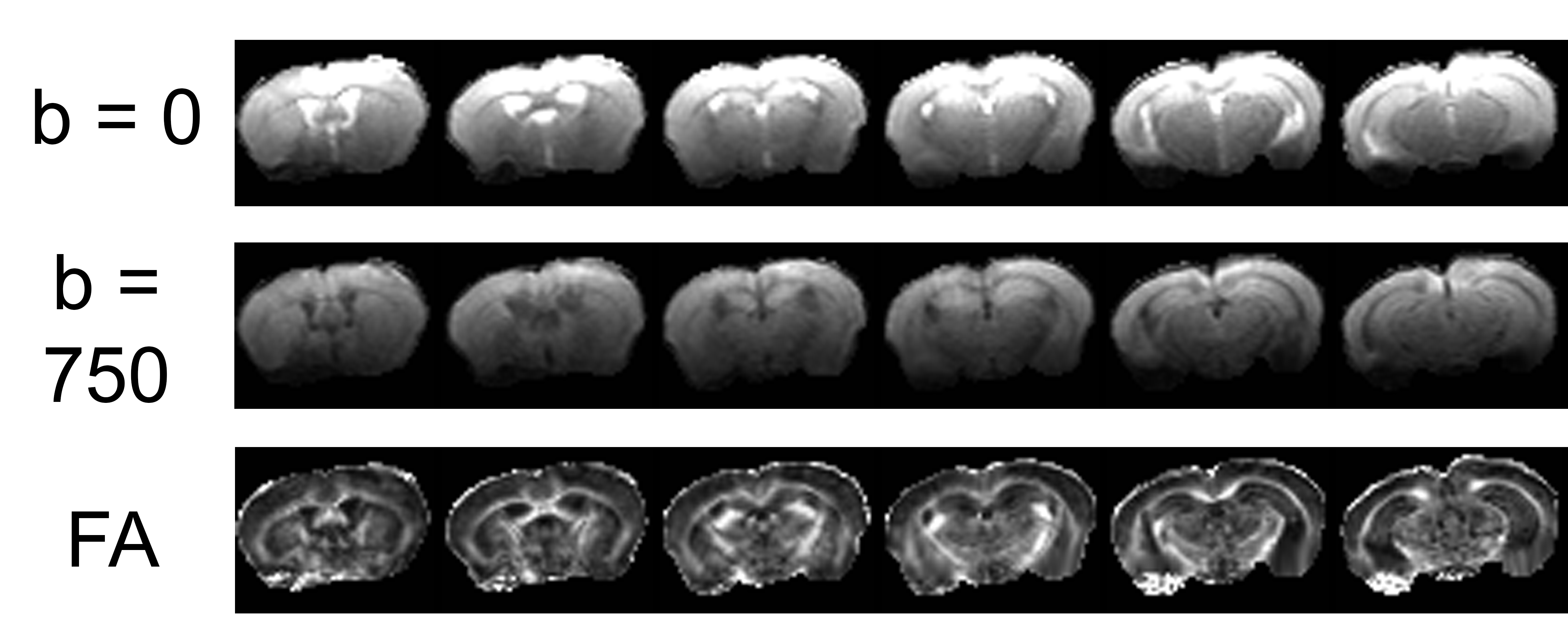

Supplement: Supplementary Figure 1 — Example of distortion-corrected data from a sham subject, demonstrating SNR. [file Image_1.PNG]

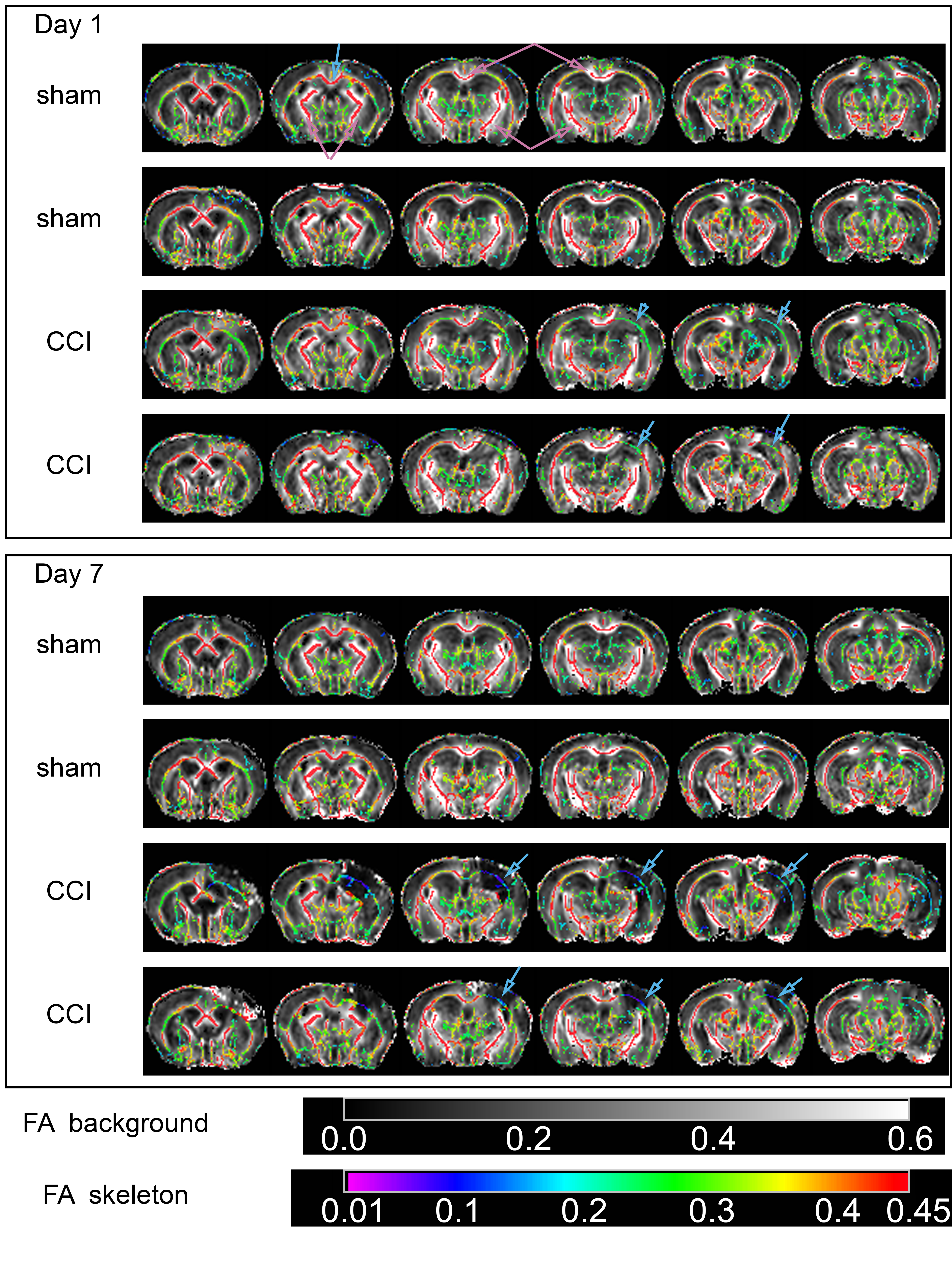

Supplement: Supplementary Figure 2 — Examples of registered FA maps from sham and CCI animals of days 2 and 7. Each individual’s registered FA maps (grayscale background) were shown with the corresponding skeletonized FA maps (rainbow scaled overlays). Generally, thinner white matter tracts (e.g., the external capsule on contralateral side) were well-matched across subjects, evidenced by the thin FA skeleton consistently covering the whole tract. Thicker white matter tracts, i.e., areas with high FA, (e.g., the corpus callosum and internal capsule, indicated on first row with purple arrows) had slight mismatches, in terms of the tract thickness and/or the FA skeleton did not always pass through the same location along the tract. TBSS skeletonizing process improve the spatial matching of these white matter tracts and overall, the skeletonizing process was accurate, i.e., the FA skeleton always fall within larger FA tracts. TBSS also reflected situation where the white matter tract was disrupted in lesion area in the CCI animals, for example the ipsilateral external capsule (indicated by light blue arrows). On the registered FA maps, the external capsule in the lesion area disappeared and the corresponding FA skeleton passing through the area, the FA value was significantly lower to than the contralateral external capsule FA skeletonized FA tract (∼0.1, below the TBSS FA threshold). The white matter tracts were grossly aligned and consequently the more homogenous (in terms of DTI metrics pattern) gray matter area was sufficiently well-matched for voxel-wise spatial statistics. This is relevant since TBSS does not provide results on DTI changes in the gray matter. [file Image_2.PNG]

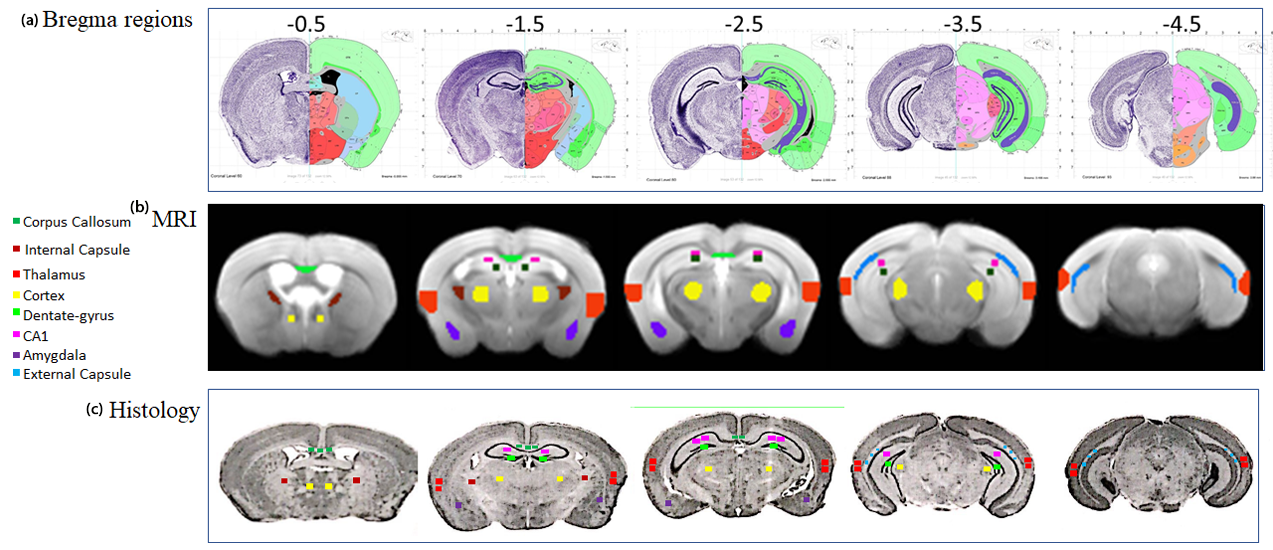

Supplement: Supplementary Figure 3 — Figure representing the sections and regions of interest chosen for region-based quantification: (A) Representative images from the developing Allen mouse brain atlas to demonstrate the bregma region (−0.5 to −4.5 mm) used for immunohistochemistry analysis and quantification. (B) Images of the MRI slices from the same bregma region were chosen to demonstrate the Regions-of-interest that were drawn manually on the study-specific template using the Australian mouse brain mapping consortium template (Watson et al., 2017) and the Allen adult mouse brain atlas (https://alleninstitute.org/) as a reference. All slices between the bregma regions −0.5 to −4.5 mm were covered in the ROI mask. (C) Representative serial coronal sections chosen for immunohistochemistry quantification. Regions of interest were Amygdala, thalamus, hippocampus (CA1 and dentate gyrus), cortex (closer to the amygdala region), corpus callosum, internal and external capsule shown with different colors on the slices. Two sample boxes (one large and one small) were used to create multiple ROIs for all the slices, that were used to quantify pathologies in different areas as shown in different colors in the figure. To be consistent with the size of the ROIs, same set of ROIs for all the five sections were used for all the animals with slight adjustment in the location if needed. [file Image_3.TIF]

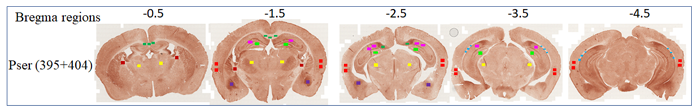

Supplement: Supplementary Figure 4 — Histology sections of a representative sham mouse stained for p-tau. [file Image_4.TIF]
