# Supplementary material for: Diffusion Tensor Imaging Detects Acute Pathology-Specific Changes in the P301L Tauopathy Mouse Model Following Traumatic Brain Injury
Source: Front Neurosci. 2021 Feb 24;15:611451. doi: 10.3389/fnins.2021.611451 (PMC7943881; doi:10.3389/fnins.2021.611451)
Supplement: Supplementary file 5 [file Data_Sheet_1.PDF]

Supplementary Material

1. Table 1S: GFAP counts

Areas	D1S	D1T	P value	D7S	D7T	P value
Ip-Amg	35.33 ± 5.158	45.56 ± 6.385	0.5307	27.06 ± 4.303	100.2 ± 5.023	0.0001*
Cn-Amg	29.39 ± 5.909	31.33 ± 5.843	0.9948	29.06 ± 5.537	41.11 ± 5.352	0.4532
Ca1	94.83 ± 9.899	79.46 ± 8.402	0.5471	84.08 ± 6.110	165.4 ± 7.474	0.0001*
DnG	92.93 ± 5.466	87.79 ± 7.060	0.9306	85.01 ± 6.438	170.5 ± 4.985	0.0001*
Ip-Cx	12.53 ± 1.356	25.44 ± 4.766	0.0463*	10.41 ± 1.532	68.56 ± 3.819	0.0001*
Cn-Cx	8.267 ± 1.390	9.867 ± 1.441	0.8812	7.133 ± 1.006	20.40 ± 2.101	0.0001*
Ip-Th	6.250 ± 1.466	23.67 ± 4.942	0.0266*	6.222 ± 1.578	111.9 ± 5.835	0.0001*
Cn-Th	7.167 ± 0.7340	12.61 ± 6.498	0.9140	7.056 ± 1.595	22.67 ± 9.727	0.2735
CC	5.537 ± 0.4948	7.555 ± 1.271	0.3452	6.482 ± 0.7949	14.57 ± 5454	0.0001*
Ip-EC	9.917 ± 0.8182	10.21 ± 0.911	0.9968	8.667 ± 1.210	14.08 ± 1.028	0.0054*
Cn-EC	10.17 ± 1.849	9.542 ± 1.081	0.9909	9.250 ± 1.884	15.50 ± 0.9704	0.0379*
Ip-IC	46.25 ± 6.199	99.00 ± 9.201	0.0009*	64.33 ± 4.897	123.00 ± 10.79	0.0003*
Cn-IC	49.50 ± 8.402	72.17 ± 9.376	0.2548	52.50 ± 9.268	68.50 ± 6.059	0.5438

2. Table 2S: GFAP-MRI correlation

Areas	FA	AD	MD	RD
Ip-Amg	0.2642; 0.0121	0.3340; 0.0039	0.3173; 0.0051	0.1840; 0.0411
Cn-Amg	-	-	-	-
Ca1	-	-	-	-
DnG	-	-	-	-
Ip-Cx	0.2884; 0.0082	-	-	-
Cn-Cx	-	-	-	-
Ip-Th	0.1598; 0.0500	0.2658; 0.0118		
Cn-Th	-	-	-	-
CC	0.2805; 0.0078	-	-	-
Ip-EC	-	-	0.2251; 0.0222	0.2609; 0.0128
Cn-EC	-	0.1997; 0.0325	-	-
Ip-IC	0.4915; 0.0001	0.3622; 0.0019	-	-
Cn-IC	-	-	-	-
